# 2-Guanidinobenzimidazole as Ligand in Supramolecular, Coordination and Organometallic Chemistry

**DOI:** 10.3390/ijms26031063

**Published:** 2025-01-26

**Authors:** Itzia I. Padilla-Martínez, Alejandro Cruz, Efrén V. García-Báez

**Affiliations:** Laboratorio de Química Supramolecular y Nanociencias, Departamento de Ciencias Básicas, Instituto Politécnico Nacional-UPIBI, Av. Acueducto s/n, Barrio la Laguna Ticomán, Ciudad de México 07340, Mexico; ipadillamar@ipn.mx (I.I.P.-M.); egarciaba@ipn.mx (E.V.G.-B.)

**Keywords:** 2-guanidinobenzimidazoles, boron compounds, metallic complexes, organometallic complexes, host–guest complexes, biological activity of complexes

## Abstract

The benzimidazole core (BI) plays a central role in biologically active molecules. The BI nucleus is widely used as a building block to generate a variety of bioactive heterocyclic compounds to be used as antihelmintics, antiprotozoal, antimalarials, anti-inflammatories, antivirals, antimicrobials, antiparasitics, and antimycobacterials. A versatile BI derivative is the 2-guanidinobenzimidazole (2GBI), which, together with its derivatives, is a very interesting poly-functional planar molecule having a delocalised 10 π electrons system conjugated with the guanidine group. The 2GBI molecule has five nitrogen atoms containing five labile N–H bonds, which interact with the out-ward-facing channel entrance, forming a labile complex with the biological receptor sites. In this work, 2GBI and their derivatives were analyzed as ligands to form host–guest, coordination and organometallic complexes. Synthesis methodology, metal geometries, hydrogen bonding (HB) interactions, and the biological activities of the complexes were discussed.

## 1. Introduction

Guanidine derivatives (R-NH)_2_C=N-R have applications as ion transporters [1], catalysts [2], chemical sensors [3,4] and anticancer drugs [5,6]. Their structure is characterised by two parallel-disposed NH moieties, which allow characteristic hydrogen bonding (HB) patterns. Guanidines form coordination complexes with transition and main group metals [7]; among them, platinum and iridium complexes stand out because of the nature of the coordination [8,9,10]. Recently, ruthenium-based complexes with guanidine-type ligands showed prominent anticancer activity [11,12,13,14,15]. Since ruthenium can bind DNA in several possible modes, ruthenium complexes have been used to treat platinum-resistant cancers with the aim to improve selectivity and ameliorate side effects and cytotoxicity [16].

**Figure 1:**
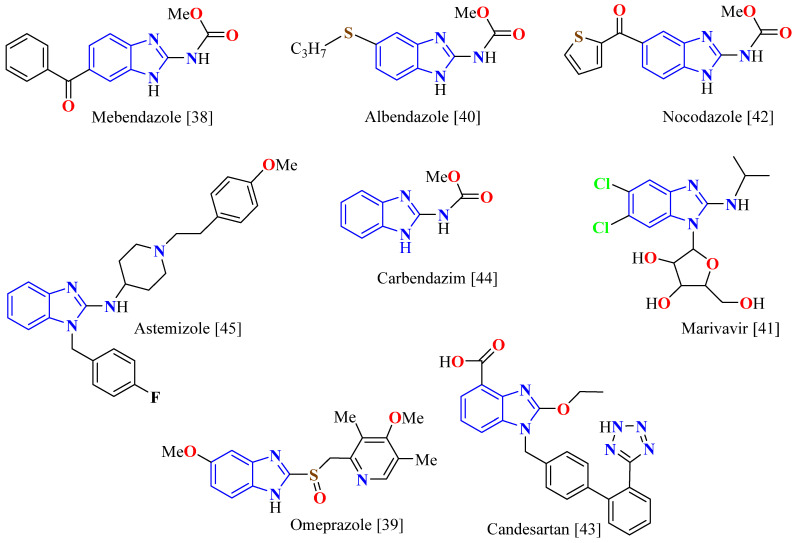
BI derivative compounds with application in medicine.

On the other hand, the synthesis of benzimidazole derivatives (BI) is of great importance due to its wide pharmacophoric spectrum. Several Bis showed biological activities as antiprotozoal [17], analgesic [18], antimicrobial [19], antihistaminic [20], anti-inflammatory [21], anticancer [22], acetylcholinesterase inhibitory [23], antimalarial [24], anti-HIV [25], antitubercular [26], and antiviral [27]. Many of them are marketed drugs sold as mebendazole (antihelmintic) [28], omeprazole (anti-ulcerative) [29], albendazole (antiprotozoal) [30], maribavir (antiviral) [31], nocodazole (anticancer) [32], candesartan (antihypertensive) [33], carbendazim (fungicide) [34], and aztemizole (antihistaminic) [35], Figure 1. Most of them, except for omeprazole and candesartan, are derived from 2-aminobenzimidazole (2ABI), which has an intra-cyclic guanidine moiety.

In 2-guanidinobenzimidazole (2GBI), the structural benefits of guanidine and BI are combined in a single molecule. This centenary heterocycle was first reported in 1921 [36], and only 10 years later, its activity as a hypoglycaemic agent was informed [37]. Moreover, further interest in this compound was renewed almost three decades later when the antituberculosis activity of several chloro-substituted-2GBI was reported [38]. Since then, 2GBI has become a ligand widely studied because of its structural features, which make it a superbase and a synthetic pharmacophore with many biological applications. In fact, only in the present year has it been demonstrated that 2GBI exerts a protective effect against LPS-induced neuroinflammation [39]. 2GBI has been identified as a potent and selective inhibitor of two folate enzymes acting against Trypanosomiasis and Leishmaniasis [40]. In addition, a new method of synthesis of 2GBI has been reported [41]; metal complexes of 2GBI (**M**-2GBz, **M**^II^ = **Zn**, **Ni**) were found to bind DNA, acting as potent antiproliferatives against MCF-7 cell line [42] as well as catalyst for C–C bond formation in the cycloaddition of epoxides and carbon dioxide [43] and multicomponent reactions [44], with very good yields and rates.

Furthermore, it has been reported that the guanidine group in 2GBI contributes to the activity of several antibiotics [45]. 2GBI is a very important molecule because its biological activities found just as a stimulator [46], inhibitor of the transport of **Na**^+^ and **K**^+^ in the apical membrane of the skin [38], diminishes the gastric acid secretion [47], acts as hypoglycaemic [48], hypotensive [49], uncoupler of photophosphorylation [50], and recently, 5-cloro-2GBI was found to be a non-selective inhibitor of the human hHv1 channel [51].

In this sense, and in continuation with our work on 2GBI [52], herein we review the use of 2GBIs as ligands in supramolecular chemistry and coordination towards elements of the main group and transition metal ions, including organometallic compounds. We offer a brief description of each bibliographic source to provide the reader with the full scope of the analyzed work and highlight the key details. The aim of this review is to provide the scientific community with an updated compendium on these aspects of the chemistry of 2GBI that serve as a starting point to expand the frontier of knowledge.

## 2. Structural Description of 2GBI

**Scheme 1:**
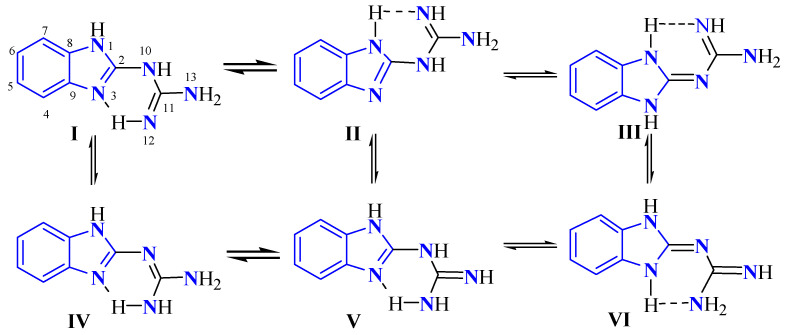
Tautomers and conformers of 2-guanidinobenzimidazole.

2GBI contains five nitrogen atoms with free lone pairs and five acid hydrogen atoms; these structural features provide them with an amphoteric character. The exchange of hydrogen atoms allows 2GBI to exist as different tautomer and conformer structures stabilised by intramolecular and intermolecular HB interactions, Scheme 1. The structure and dynamic behavior of 2GBI in solution have been studied by ^1^H-, ^13^C- and ^15^N-NMR spectroscopy [53,54,55,56]. The equivalent tautomers **I** and **II**, containing an intramolecular HB six-membered ring N12–H∙∙∙N3, were the main contributors [56]. The single crystal X-ray diffraction studies confirmed the formation of this HB; the guanidine group is bent by 13.8° to the BI plane, and the remaining NH hydrogen atoms are involved in additional intermolecular HB, which connect the molecules in a complex three-dimensional network [57,58]. A theoretical study (RHF/6-321, NBO) revealed that the stabilisation energy of the six-membered HB ring was calculated at 22.6 kcal/mol and was found to be in resonance between the two contributors [59].

Furthermore, 2GBI was reacted with Brönsted acids and methyl iodide to obtain information about 2GBI Lewis base sites by NMR and X-ray diffraction techniques, Table 1 [56]. For protonation and methylation reactions, imidazole N3 is preferred as the basic site in solution, and diprotonation at both N3 and N10 occurs under strongly acidic conditions.

## 3. Host-Guest Complexes and Supramolecular Assemblies

**Figure 2:**
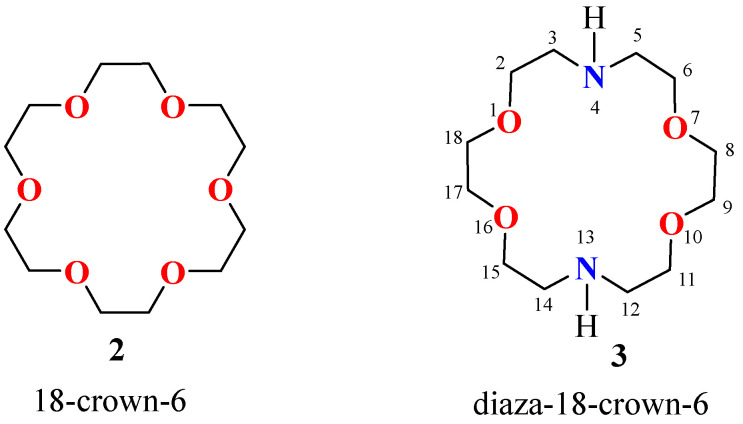
Crown ethers as host for 2GBI molecules.

The single-crystal X-ray structures of 2GBI complexes with the ethers 18-crown-6, **2**, and diaza-18-crown-6, **3** were described. The host–guest complexes were formed with 2:1 stoichiometry, (2GBI)_2_-**2** and (2GBI)_2_-**3**, Figure 2 [60]. In both host–guest complexes, two 2GBI guest molecules approach from both sides of the plane of the crown ether ring, sterically fitting with NH_2_ and NH∙∙∙OH_2_-functional groups and binding by multiple NH∙∙∙O and OH∙∙∙O HBs.

**Figure 3:**
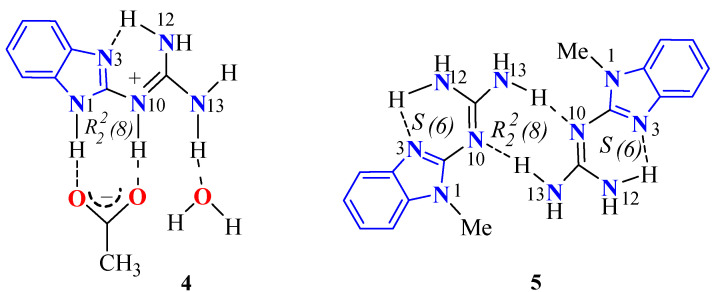
Supramolecular assemblies of 2GBIium acetate **4** and *^N^*Me-2GBI **5**.

Crystals of salt **4** were precipitated from a mixture of 2GBI and acetic acid in water. 2GBIium acetate monohydrate **4** crystallised as supramolecular assembly. The structure showed that N10 was protonated instead of the protonation at N3 in solution, Figure 3 [56]. The oxygen atoms of the acetate group and a water molecule were linked through HBs to the hydrogen atoms on N1, N10 (R^2^_2_(8), and N13 of the 2GBIium ion in a planar conformation in DDD–AAA triads. In addition, an intramolecular N12–H···N3 HB, S(6), was observed. On the other hand, the *^N^*Me-2GBI was synthesized and recrystallised from a 30:70 mixture of CH_2_Cl_2_–H_2_O, and after five months, brown crystals were formed. The X-ray diffraction analysis showed the supramolecular assembly **5**, whose X-ray diffraction showed a dimeric array stabilised by two intermolecular HBs between N13–H∙∙·N10 (2.02–2.03 Å) and an intramolecular HB N12–H···N3 with a bond length of 2.0 Å, forming R^2^_2_(8) and S(6) ring motifs, respectively, which are common to both **4** and **5**.

**Figure 4:**
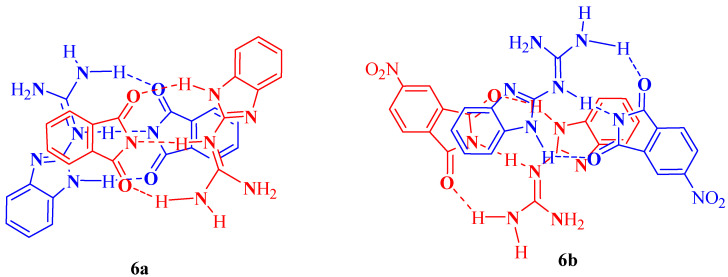
2GBI—Phthalimide stacking patterns in **6a** and **6b** arrays supramolecular assemblies.

Crystals were precipitated from a solution mixture of 2GBI and phthalimide. The X-ray and neutron diffraction analyses showed the supramolecular assembly **6a** was formed. The structure showed a short HB, N10–H∙∙∙N [2.692(4) Å], Figure 4 [61], where one proton of the phthalimide was transferred to 2GBI. The resulting ionic pair was stabilised by π-stacking interactions (3.3 Å of interplanar distance). This structure was compared with that of the supramolecular assembly between 2GBI and 4-nitrophthalimide, obtained as a bright orange crystalline precipitate **6b** in 80% yield. In most tautomers, N3, N10, and N12, featuring the N–H bonds, were responsible for HB interactions in the supramolecular assembly.

**Figure 5:**
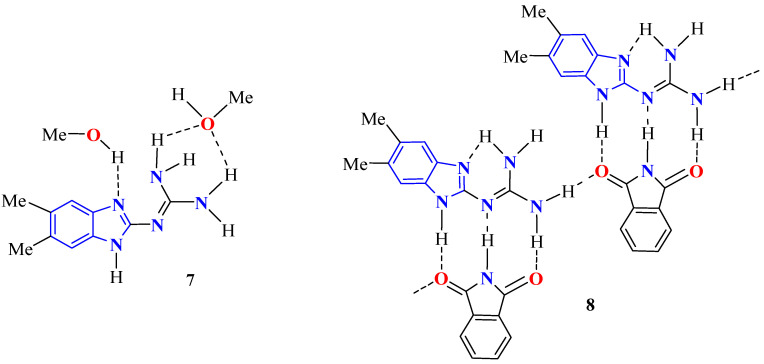
Me_2_2GBI:MeOH **7** and Me_2_2GBI:phthalimide **8** supramolecular assemblies.

The supramolecular assemblies formed by a solution mixture of N″-(5,6-dimethyl-1H-benzimidazol-2-yl)-guanidine (Me_2_2GBI) with methanol **7** and phthalimide **8** were analyzed by X-ray diffraction and compared each other, Figure 5 [62]. The supramolecular assembly **8** had complementary DAD/ADA HB motifs to form dimers. In addition, the dimers were linked into chains by the formation of O–H∙∙∙N HBs. The steric demand of the Me groups inhibits the formation of a more structured crystal network.

**Scheme 2:**
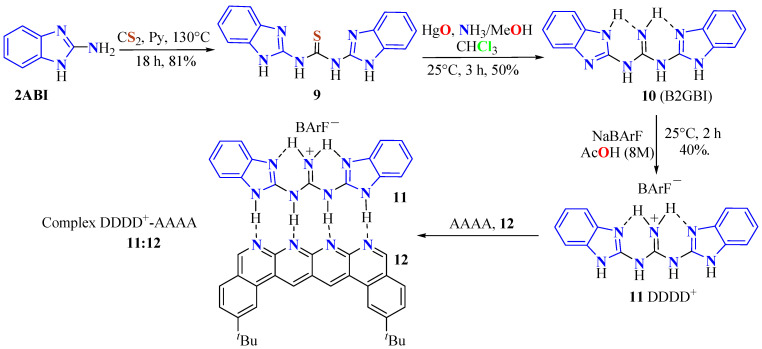
Synthesis of bis-2GBI **10** and the molecular assembly DDDD-AAAA **11**:**12**. BArF^−^ = [B(3,5-(CF_3_)_2_C_6_H_3_)_4_].

Bis-guanidinobenzimidazole (BGBI) **10** is obtained from **9**, as depicted in Scheme 2. After protonation with HBArF (tetrakis [3,5-bis(trifluoromethyl)phenyl]boric acid) to form **11**^+^BArF^−^ and reaction with the tetrapyridine **12**, the quadruple N–H···N DDDD^+^–AAAA molecular assembly **11**^+^:**12** was formed [63]. The assembly **11^+^**:**12** exhibited excellent stability for an HB array in a range of solvent systems, with association constants (K_a_) values of 3 × 10^12^ M^−1^ in CH_2_Cl_2_, 1.5 × 10^6^ M^−1^ in CH_3_CN and 3.4 × 10^5^ M^−1^ in 10% DMSO/CHCl_3_. The ease of synthesis and strength of binding of this HB supramolecular assembly make it a promising candidate for incorporation into supramolecular polymers and other materials.

## 4. 2GBI Coordination Complexes of the Main Group Elements (B, Sn and Sb)

**Scheme 3:**
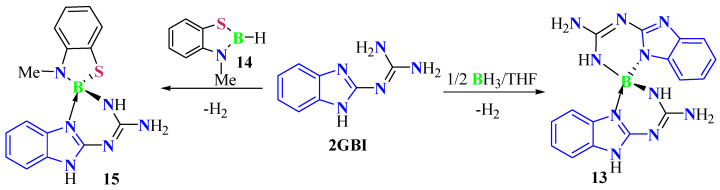
Boron coordination complex spiro-cycles **13** and **15** from 2GBI.

2GBI complexes of the main group elements are scarce; only boron, tin and antimony complexes are known. The spiro–boron coordination complexes **13** and **15** were synthesized from 2GBI and the borohydride **14** or borane, respectively, Scheme 3 [64]. In both compounds, 2GBI acted as a bidentate ligand; the pyridine-like nitrogen N3 coordinates to the boron atom, whereas the exocyclic N12 forms a covalent bond shaping a six-membered ring. The boron acquires a negative charge, whereas the positive charge in 2GBI is delocalised between nitrogen atoms. This bonding pattern is common to all boron–2GBI complexes.

**Scheme 4:**
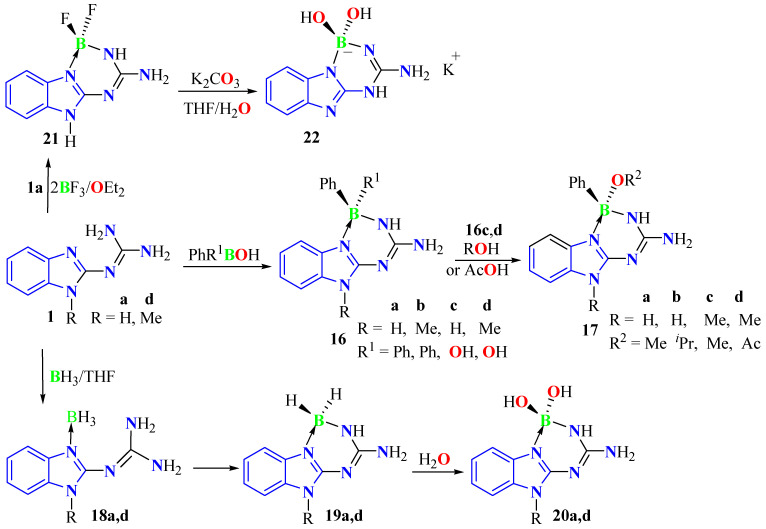
Boron coordination complex heterocycles **16**–**22** derived from 2GBI **1a** and *N*1-Me2GBI **1d**.

2GBI **1a** and the *^N^*Me-2GBI **1d** were reacted with diphenylborinic and phenylboronic acids to produce the corresponding boron coordination complexes **16a**-**d**, Scheme 4 [65]. The structures of **16a**–**d** were assigned by ^1^H, ^13^C, ^15^N and ^11^B NMR spectra and X-ray diffraction analysis. Boron complexes **16c** and **16d** were reacted with methanol, isopropyl alcohol, and acetic acid in refluxing toluene to yield the boron complex esters **17a**–**c** and the anhydride boron complex **17d**, respectively. Also, compounds **1a**,**d** were reacted with BH_3_/THF at room temperature to form the corresponding N–BH_3_ adducts **18a** and **18d**. These adducts, on heating, were transformed to the boron complex heterocycles **19a**,**d,** which, after hydrolysis, afforded the neutral dihydroxy–borate complexes **20a**,**d**. However, the reaction of 2GBI **1a** with two equivalents of BF_3_–OEt_2_ afforded the boron complex heterocycle **21** in a mixture with the 2GBIium tetrafluoroborate derivative. After purification, compound **21** was reacted with K_2_CO_3_ in a THF–water solution to give the potassium salt of the dihydroxy–borate complex **22** as colourless crystals.

**Scheme 5:**
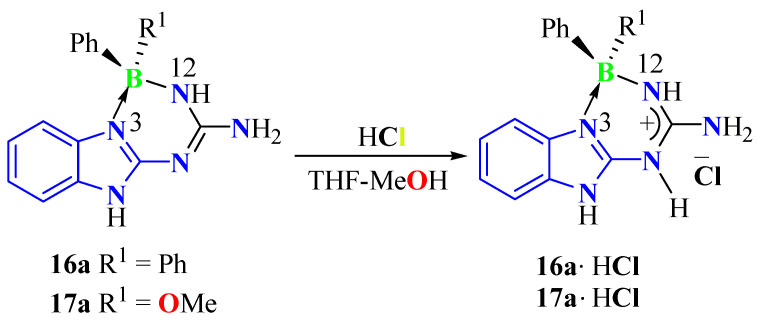
Hydrochlorides of 2GBI–boron coordination complex heterocycles from 2GBI–boron heterocycles.

The basic properties of the boron complex heterocycles **16a** and **17a** were evaluated for preparing the hydrochlorides **16a∙HCl** and **17a∙HCl**, Scheme 5. Compound **17a∙HCl** was studied by X-ray diffraction analysis [56].

**Figure 6:**
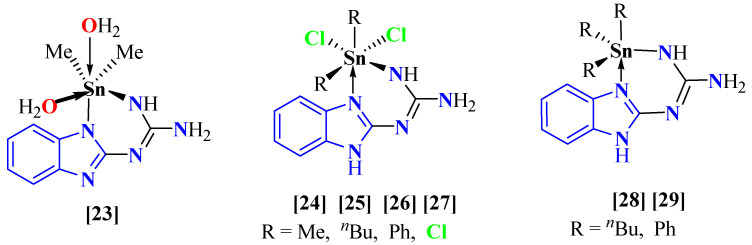
Tin complex heterocycles derived from 2GBI.

In contrast to boron coordination complex heterocycles, tin(IV) complexes are more varied in coordination modes. For instance, tin coordination complex heterocycles **[23]–[29]** were prepared from the reaction of 2GBI with R_2_**Sn**Cl_2_ or R_3_**Sn**Cl, Figure 6 [56,66]. The ^119^Sn NMR spectrum of the tin complex **[23]** showed a single signal at δ −202.9 ppm, characteristic of a hexacoordinated tin atom, where 2GBI was deprotonated at N1 and N12 and acting as a bidentate ligand. On the other hand, tin complexes **[24]–[27]** were suggested to be hexacoordinated and the tin complexes **[28]–[29]** in pentacoordinated geometries. In both cases, 2GBI acts as a bidentate monoanionic ligand, deprotonated at N12. The mass spectrometry (FAB) showed complexes **[24]–[27]** associated with chlorine bridges.

**Scheme 6:**
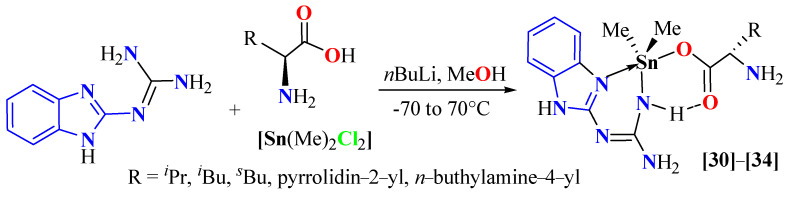
Dimethyl tin complex heterocycles derived from 2GBI and amino acids.

Five dimethyl tin coordination complex heterocycles **[30]–[34]** derived from 2GBI and several amino acids were synthesized in 96–98% yield, Scheme 6 [67]. The *L*-amino acids used were valine, leucine, isoleucine, proline, and lysine. The tin complexes were characterised by ^1^H and ^13^C NMR spectra and mass spectrometry, as well as UV–visible spectroscopy and microanalysis.

**Scheme 7:**
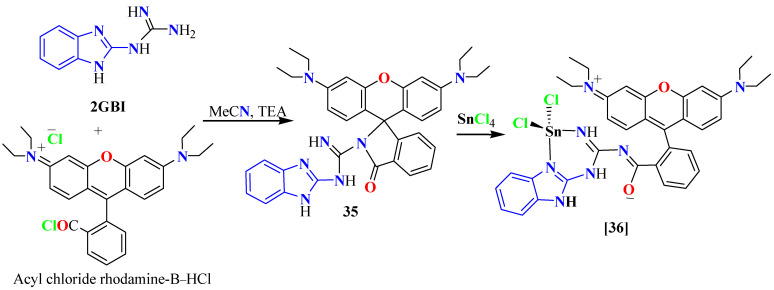
Synthesis of a functionalyzed rhodamine-2GBI **35** for detection of tetravalent tin.

The ligand rhodamine-2GBI **35** was synthesized in 80% yield by amidation of the acyl chloride of rhodamine B hydrochloride with 2GBI, Scheme 7. The ligand **35** reacted with tin tetrachloride to afford the tin complex heterocycle **[36]**. The rhodamine-ligand **35** was used for the detection of tetravalent tin in environmental, geological, and biological samples with excellent selectivity, sensitivity, photostability and anti-interference performance. The authors confirmed the 1:1 stoichiometry between **35** and Sn^IV^ by Job’s plot and mass spectrum, with an association constant of 2.99 × 10^6^ M^−1^, as well as the reversibility of the process. The structure of the tin coordination complex **[36]** was proposed through theoretical calculations. However, as proposed, it corresponds to the Sn^II^ complex, which the authors do not discuss any further [68].

**Figure 7:**
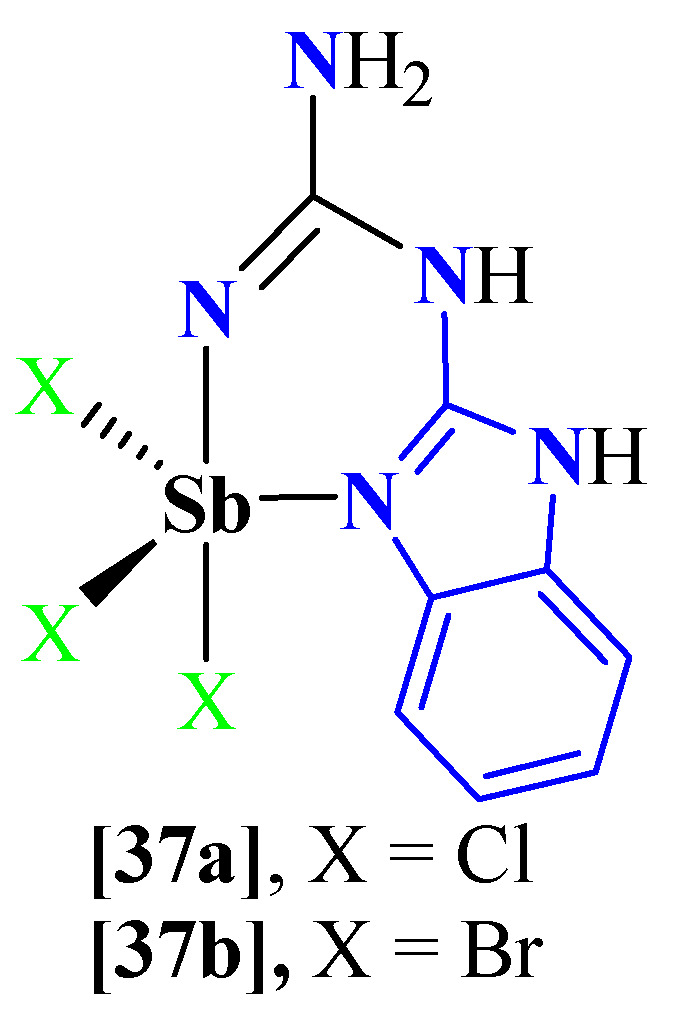
[**Sb**(2GBI)X_3_] complexes **[37a,b]**.

Antimony complex heterocycles **[37a,b]** of formula **[Sb**(2GBI)X_3_**]** (X = Cl, Br), Figure 7, were prepared by direct reaction between 2GBI and the **Sb**X_3_ salt. The antimony complex heterocycles were pentacoordinated with square-pyramidal geometry, where 2GBI behaves as a bidentate ligand with the N donor atom of the BI group sharing the plane with the **Sb**X_3_ fragment and the N donor atom of guanidine moiety in the apical position [69].

## 5. Transition Metal Coordination Complexes

Indian research groups were pioneers in the formation of transition metal coordination complexes of 2GBI. They investigated the behavior of 2GBI as monodentate and bidentate ligands coordinated towards **M**^II^ metal ions (**M**^II^ = **Cu**, **Co**, **Zn** and **Ni**) **[38]–[44]**. The formula of these transition metal complexes was proposed based on elemental analysis, as listed in Table 2 [70]. In general, the transition metal complexes were formulated with stoichiometries 1:1 or 1:2, **M**:2GBI.

**Figure 8:**
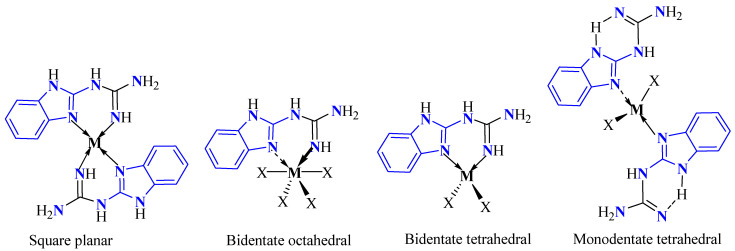
Coordination spheres of 2GBI ligands in **M**^II^ complexes (**M**^II^ = **Cu**, **Co**, **Zn, Ni**).

Later, the transition metal coordination species **38**–**42** and **44** were revisited from a structural point of view [71]. The structure of the **Ni**^II^ complex **[44]**(NO_3_)_2_ was analyzed by X-ray diffraction to confirm the 1:2 stoichiometry and the coordination of 2GBI through N3 and N12 in square planar geometry. In all **M**^II^ (**M** = **Cu**, **Zn**, **Ni**) and **[40**C1_2_(H_2_O)_2_**]**∙5H_2_O complexes, 2GBI is coordinated in bidentate fashion through N3 and N12, as found in the complex **[44]**(NO_3_)_2_. In the case of the **Cu**^II^ complexes **[38**Cl_2_**]**, **[38**Br_2_**]**, and **Zn**^II^ complexes **[42**C1_2_**]**∙H_2_O, and **[42**Br_2_**]**∙H_2_O, the structures are tetrahedral in contrast to the octahedral **Co**^II^ complex [**40**C1_2_(H_2_O)_2_]∙5H_2_O containing two coordinated water molecules. The structures with two 2GBI bidentate ligands in the of **Ni**^II^ complexes **[44**Cl_2_**]**·H_2_O, **[44**Br_2_**]**·3H_2_O, **[44]**NO_3_, **[44]**(AcO)_2_, and **Cu**^II^ complexes **[39**Br_2_**]**·2H_2_O, **[39]**(NO_3_)_2_·H_2_O, **[39]** (AcO)_2_·H_2_O have a square-planar geometries. A monodentate coordination mode of 2GBI was proposed through imidazole N3 in a tetrahedral geometry for **Co**^II^ complexes of 1:2 stoichiometry, Figure 8.

The **Co**^II^ complexes derived from 2GBI: **[41]**X_2_ (X^−^ = NO_3_, SO_4_, Br and I) were synthesized, and their thermal behavior was studied using the thermogravimetric analysis (TGA) and differential thermal analysis (DTA) [72]. Complete decomposition of the chelates was generally accomplished in two stages, and this set of complexes showed comparable thermal stability.

On the other hand, the structure of the ionic zinc complex **[43]**(NO_3_)_2_**∙**H_2_O was obtained after a week as a crystalline precipitate from the reaction of 2GBI with **Zn**(NO_3_)_2_·H_2_O. The X-ray diffraction showed two 2GBI ligands coordinated to **Zn**^II^ through N3 and N12 atoms, forming a tetrahedral geometry with two nitrates outside the coordination sphere [73].

The diamagnetic rose-red **Ni**^II^ complex **[44]**I_2_·2H_2_O was prepared. The complex lost two molecules of water when heating between 60–100 °C without any change in colour. The complex was heated until 210 °C without any change. However, when heating between 208–210 °C, the substance became green and paramagnetic with the composition **[44**I_2_**]**. The Magnetic moment measurements (μ_eff_ = 3.42 B.M. at 303 °K) suggested that heating caused the breakdown of the crystal structure to produce a supramolecular polymer with octahedral geometry around the **Ni**^II^, involving interactions with the iodide ions, but from the electronic spectra it appears to have a tetragonal (D_4*h*_) structure [74].

A low-temperature single-crystal X-ray study of the **Cu**^II^ complex **[39]**(ClO_4_)_2_·H_2_O allowed a comparison of the impact of the different electron configurations on the overall cation geometries when compared with previous studies of the **Ni**^II^ complex **[44]**(NO_3_)_2_ and the **Zn**^II^ complex **[43]**(NO_3_)_2_ [75].

2GBI was used as a ligand to remove **Co**^II^ and **Zn**^II^ ions from contaminated water samples [76]. In the analysis, two 2GBI bidentate ligands combine with each metal to yield the complexes **[41]**(NO_3_)_2_ and **[43]**(NO_3_)_2_, respectively, as stable complexes which were separated in a solid phase extraction. The retained analytes were recovered to be quantified by flame atomic absorption spectrometry. The effects of other cationic and anionic interferences were considered in the quantitative analysis. The limits of detection of this method were 1.62 and 0.26 ng mL^−1^ for **Co**^II^ and **Zn**^II^, respectively.

**Figure 9:**
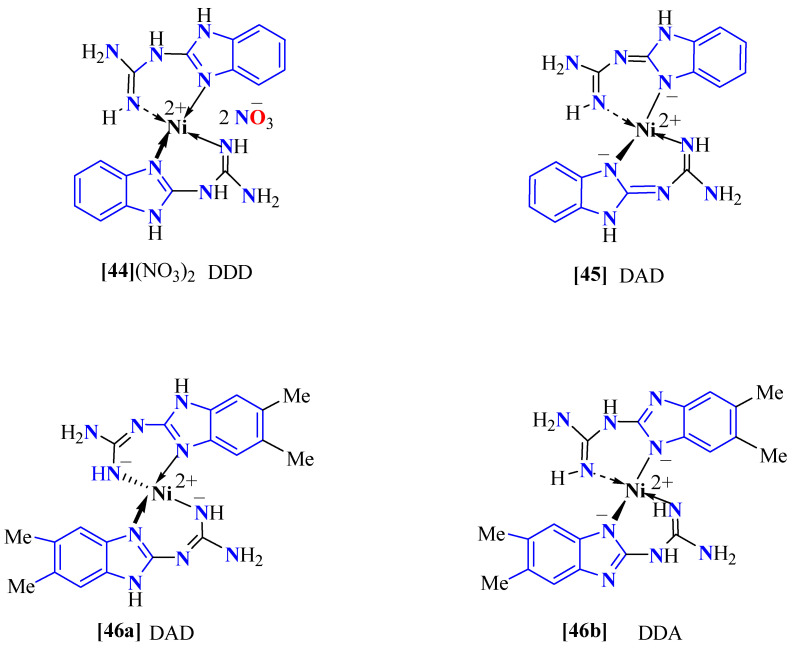
Structure of the tetrahedral bis-chelate nickel(II) complexes and their hydrogen bonding patterns. D = hydrogen bonding donor, A = hydrogen bonding acceptor.

The synthesis of complex **[44]**(NO_3_)_2_ in aqueous ammonia yields, after recrystallisation from DMSO solution, the complex in the neutral form **[45]** as a mixed solvate of formula **Ni**(**2GBI**)_2_·DMSO·½H_2_O (**[45]·**DMSO·½H_2_O), Figure 9. In contrast to the di-cationic species, the neutral complex existed in several tautomeric forms that self-assembly to form an extended supramolecular lattice stabilised by HB between the anionic 2GBIs, forming channels occupied by DMSO and water molecules when present. The tautomer of 2GBI^−^ present in **[45]·**DMSO·½H_2_O, restored much of the original HB capability of the un-complexed ligand, but different HB motifs were found [77].

**Figure 10:**
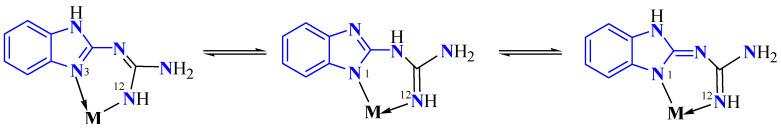
Tautomers of 2GBI^−^ in transition metal coordination complexes.

The nickel(II) complex [**Ni**(Me_2_**2GBI**)_2_]·½H_2_O (**[46]** ½H_2_O) was prepared as a dark-red solid in 41% yield, following the reported procedure as for **[45]**DMSO∙½H_2_O. The complex crystallised from DMSO as the 1:1 mixture of tautomers **[46a,b]**(DMSO)_2_; in both tautomers, the NiN_3_C_2_ units adopt a distorted square planar geometry, Figure 9. Formally, the N12 proton is substituted by the metal atom and coordinated by N3 in **[46a]**, whereas the N1 proton of BI is substituted by the metal and coordinated by N12 in **[46b]**, which, after exchanging the proton between N3 and N2, results in the tautomer **[45]**, Figure 10 [62]. Each tautomer features a characteristic hydrogen bonding pattern, such as DDD, DAD, or DDA, as indicated in Figure 9.

2GBI complexed with **M**^III^ have also been obtained. A series of twenty one **Rh(2GBI)_2_**^3+^ **47**, **Rh**(**2GBI**)_3_^3+^ **48** and **Ir**(**2GBI**)_2_^3+^ **49** complexes were prepared by the use of RhCl_3_·3H_2_O or (NH_4_)_2_**Ir**Cl_6,_ having the general formula **[M**(**2GBI**)_n_X_m_Y_o_**]**^3−m^ (**M** = **Rh**, **Ir**; n = 2,3; X^−^ = CI, Br, I, NCS, NO_3_; m = 2,3; Y = H_2_O, NH_3_ or pyridine; o = 1,2), Table 3 [78]. Hetero-chelates of the type **[M**(**2GBI**)_2_(AA)**]**^3+^ (AA = 2,2′-bipyridyl or 1,10-phenanthroline), **Rh**(**2GBI**)_2_(pic’) (pic’ = picolinate ion) were also prepared [79].

The **Cr**^III^ coordination complexes derived from 2GBI: **[Zn**(en)_2_**][Cr**(**2GBI**)(NCS)_4_**].**5H_2_O **50**, **[Co**(en)_2_(NO_2_)_2_**][Cr**(**2GBI**)(NCS)_4_**]**·H_2_O **51** and the acid complex H**[Cr**(**2GBI**)(NCS)_4_**]**·2H_2_O **52** were prepared as anionic salts from potassium hexa-thiocyanate-chromate, **K**_3_**[Cr**(NCS)_6_**]** [79]. Two NCS^−^ anions were replace by **2GBI** and **[Zn**(en)_2_**]**^2+^ and **[Co**(en)_2_(NO_2_)_2_**]**^+^ acting as counterions. The **Co**^III^ complex **[Co**(**2GBI**)(trien)**]**Cl_3_∙2H_2_O **53** (where trien = triethylenetetramine) was also synthesized in ~40% yield [80], all complexes were characterised as octahedral mononuclear complexes.

**Figure 11:**
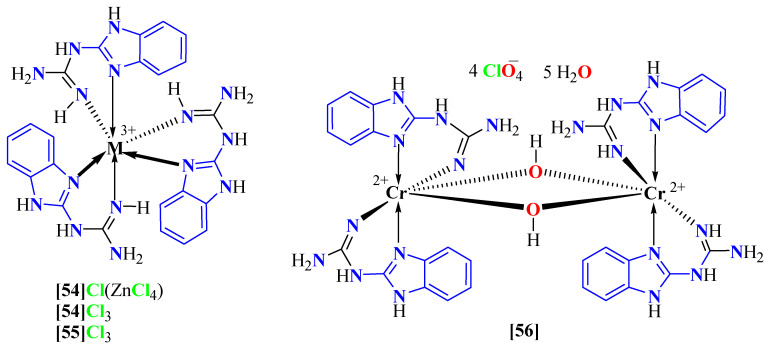
Hexacoordinated chromium and cobalt cation complexes **[54]**, **[55]** and dichromium cation complex **[56**] derived from 2GBI.

Hexacoordinated **[Cr**(**2GBI**)_3_**]**^3+^ **54**, and **[Co**(**2GBI**)_3_**]**^3+^ **55** complexes were synthesized as **[54]**Cl(**Zn**Cl_4_), **[54]**Cl_3_ and **[55]**Cl_3_ from 2GBI and the corresponding **M**^III^ salts in alcoholic solution, Figure 11 [80]. The coordination complex **[54]**Cl(**Zn**Cl_4_) precipitated as a pink solid from the reaction of 2GBI in methanol with a previously prepared suspension of anhydrous **Cr**Cl_3_ and **Zn** powder. The coordination complex **[54]**Cl_3_ precipitated as a pink solid from the reaction of anhydrous **Cr**Cl_3_ placed on Zn–Hg amalgam in methanol with a solution of 2GBI in anhydrous methanol. The coordination complex **[55]**Cl_3_ precipitated as a solid when a solution of **[Co**(NH_3_)_4_**]**(CO_3_)NO_3_ was suspended in methanol, and HCl was reacted with 2GBI in methanol. In the same conditions, the solvates **[54]**Cl(**Zn**Cl_4_)·CH_3_OH and **[54]**Cl_3_·4H_2_O as pink solid in 95% and red crystals in 2.4%, respectively, were also obtained [81].

The dinuclear coordination complex **[Cr**_2_(**2GBI**)_4_(μ^−^OH)_2_**]**(ClO_4_)_4_·5H_2_O **[56]** was formed by the reaction between **Cr**Cl_2_ with 2GBI in a deoxygenated solution of 2.0 M HClO_4_-methanol solution, after the addition of NaClO_4_ solution, Figure 11. The X-ray diffraction analysis of this coordination complex showed two bidentate ligands bonded to each **Cr**^III^ in equatorial-axial position and two O–H bridges, forming a four-membered ring. The **Cr**–O and **Cr**⋯**Cr** distances were 1.960 Å and 3.085 Å (Σ*r*_vdw_ = 4.1 Å). The strong intermolecular HBs with water molecules and perchlorate anions forced the guanidine moiety to be out of the BI plane. The **Cr**⋯**Cr** short distances are favoured by π-stacking between the BI rings (3.46 Å) [82].

Four **Co**^III^ hexacoordinated coordination complexes of formula **[57]**Cl_2_(NO_3_)·H_2_O, **[57]**Cl(NO_3_)_2_·H_2_O, **[57]**Cl_3_·3H_2_O and **[57]**(Cl_3_CCO_2_)_3_·H_2_O, where **[57]** is **[Co**(**2GBI**)_2_**]**^3+^, were synthesized from the reaction of 2GBI with **[Co**(NH_3_)_4_CO_3_**]**NO_3_ or **Na**_3_**[Co**(CO_3_)_3_**]** · 3H_2_O or **Co**Cl_3_ as diamagnetic and stable solids [83]. In addition, three magnetically non-equivalent 2GBI bidentate ligands coordinated to **Co**^III^ were found in a distorted octahedral cation complex **[57]**, and only the *mer* isomer was formed.

**Figure 12:**
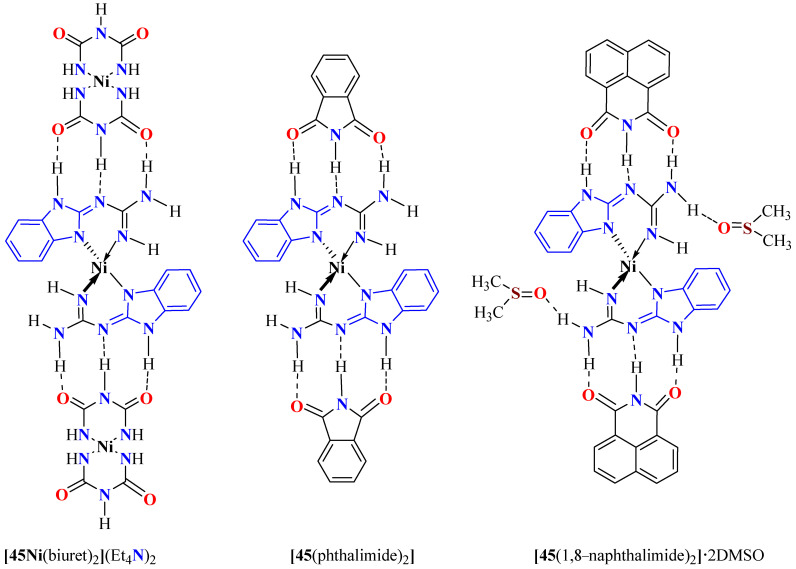
Three supramolecular assembly array complexes on the ligand of complex **[45]**. Et_4_N^+^ cations are omitted for clarity in **[45Ni**(biuret)_2_**]**(Et_4_N)_2_.

The donor-acceptor-donor (DAD) HB supramolecular assembly arrangement on 2GBI ligand of complex **[45]** was linked to (Et_4_N)_2_**[Ni**(biuret)_2_**]** units, or to 1,8-naphthalimide or phthalimide molecules, which incorporate a complementary acceptor-donor-acceptor (ADA) HB supramolecular assembly arrangement to form the corresponding three supramolecular assembly array complexes (Et_4_N)_2_**[Ni**(biuret)_2_**]**:**[45]**, **[45]**:(phthalimide)_2_ and **[45]**:(1,8-naphthalimide)_2_·2DMSO, Figure 12 [77,84]. The geometry around the metal ion and the nature of the formed HB network had significant influences on the supramolecular assembly structure adopted. An interesting combination of intramolecular HB and close π-stacking interactions also occurs in each species.

As a yellow solid, the **Fe**^III^ complex **[Fe**(**2GBI**)_3_**]**^3+^ **[58]** was synthesized as **[58]**(NO_3_)_3_ from 2GBI and **[Fe**(DMSO)_6_**]**(NO_3_)_3_ [85]. The electron transfer reactions between plastocyanin (PC) with this complex and the previously synthesized **Cr**^III^ complexes **[54]**(Cl)(**Zn**Cl_4_), **[54]**Cl_3_, **Co**^III^ complexes **[55]**Cl_3_, **[55]**Cl_2_NO_3_, **[55]**(CCl_3_CO_2_)_3_ and dichromium complex **Cr**_2_(**2GBI**)_4_(OH)_2_(ClO_4_) **[56]** were studied. The **Fe**^III^ complex **[58]**(NO_3_)_3_, oxidised PC following a simple second-order outer sphere mechanism with rate constants for electron transfer of 1.4 × 10^4^ ± 1.1 × 10^2^ and 706.2 ± 12.7 M^−1^ s^−1^, respectively. The **Co**^III^ and **Cr**^III^ complexes inhibited the electron transfer process.

**Figure 13:**
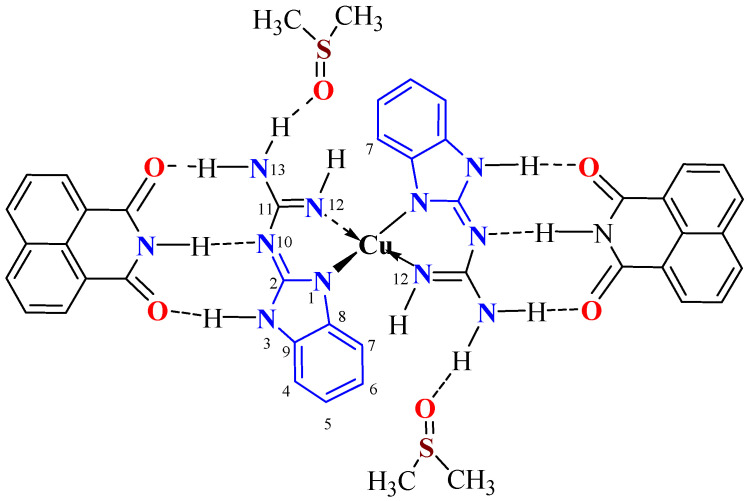
Assembly of **[59]**:(1,8-naphthalimide)_2_·2DMSO.

The addition of an ammoniacal solution of CuCl_2_ to 2GBI in methanol afforded the neutral coordination complex **[Cu**(**2GBI**)_2_**] [59]**, [86]. The complex formed supramolecular assemblies with 1,8-naphthalimide in hot DMSO based on DAD HB supramolecular motifs. In the X-ray crystallography of the **[59]**:**[**1,8-naphthalimide**]**_2_ · (DMSO)_2_ molecular assembly, Figure 13, a planar ligand was observed in a distorted tetrahedral square planar geometry around the **Cu**^II^. The molecular assembly cannot be planar due to the steric repulsion of the hydrogen atoms on C7 and N12 of opposite ligands. The structure was comparable with the analogous **Ni**^II^ complex **[45]**:(1,8-naphthalimide)_2_·2DMSO previously reported.

**Figure 14:**
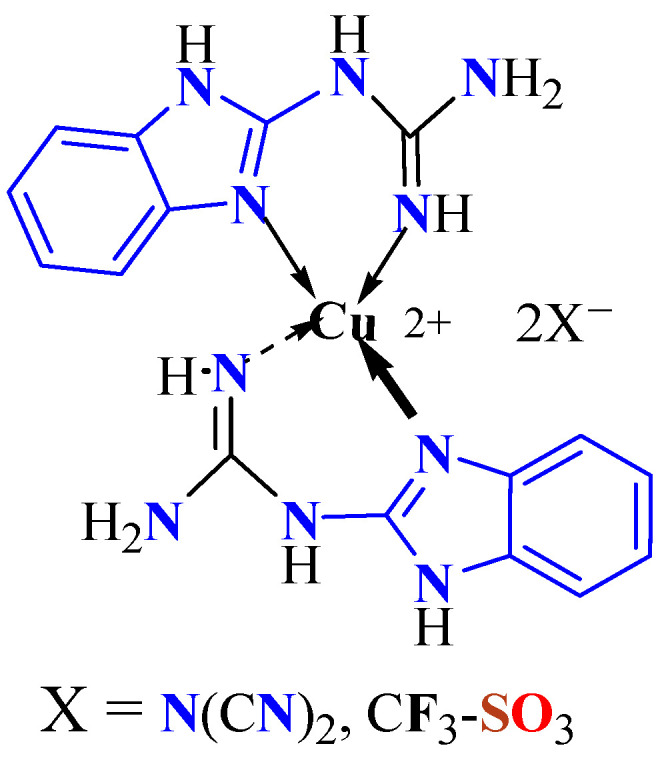
Cu^II^ complexes **[39]**(N(CN)_2_)_2_ and **[39]**(CF_3_SO_3_)_2_.

The **Cu**^II^ coordination complexes **[39]**(N(CN)_2_)_2_ and **[39]**(CF_3_SO_3_)_2_ were synthesized to analyse their X-ray diffraction structure, Figure 14. Two 2GBI bidentate ligands coordinated to the **Cu**^2+^ cation in a tetrahedral distorted square-planar geometry were observed in **[39]**(N(CN)_2_)_2_ [87]. The **Cu**^II^ and the dicyanamide anions NC-N-CN^−^ lie on a two-fold axis and are linked via N–H∙∙∙N hydrogen bonds, forming a two-dimensional network parallel to the (010) direction.

In contrast, the structure of **[39]**(CF_3_SO_3_)_2_ showed the presence of two isomeric coordination complexes, a purple α-**[39]**(CF_3_SO_3_)_2_ and a green β-**[39]**(CF_3_SO_3_)_2_. In both complexes, the **Cu**^II^ ion adopted a distorted tetrahedral geometry with torsion angles at around 40°. The position of the triflate anions was different between the isomers, therefore, the hydrogen bonding network [88].

**Figure 15:**
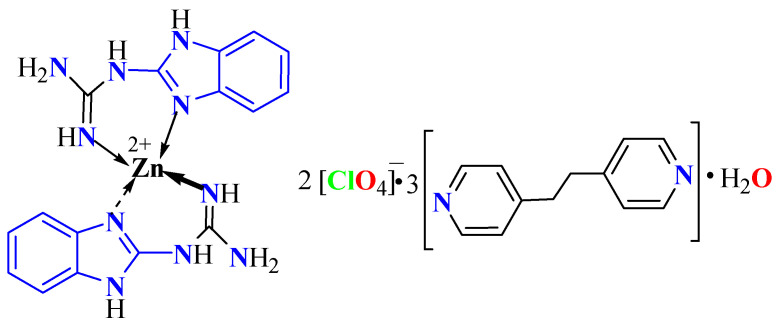
Structure of the one-dimensional (1D) supramolecular complex **[43]**(µ-bpe)_3_(ClO_4_)_2_·H_2_O.

While a solution of 2GBI and zinc chloride in methanol allowed the formation of the coordination complex **[43]**(NO_3_)_2_, a solution of 2GBI with NaClO_4_, **Zn**(Ac)_2_·2H_2_O and 1,2-bis(4-pyridyl)ethylene (bpe) afforded the complex **[43]**(µ-bpe)_3_(ClO_4_)_2_**^∙^**H_2_O, Figure 15 [89]. The X-ray diffraction analysis showed a one-dimensional polymer complex involving macrocycle rings as the result of non-covalent bridging bpe ligands via N–H∙∙∙N and N∙∙∙N interactions, N–H∙∙∙bpe∙∙∙bpe∙∙∙H–N, with the basic repeating **[43]**(µ-bpe)_3_ClO_4_·H_2_O units connecting **[43]** nodes. The calcination of this complex was used to produce **Zn**O nanoparticles.

In the field of nanomaterials, 2GBI molecules were anchored with poly-pyrrole (PPy) nanowires to form the supramolecular assembly GBI@PPyNWs. This material was used for sensitive and selective fluorescence detection of **Fe**^III^ based on the formation of a coordination complex [90]. It is noteworthy that the complexation in water increased the fluorescence intensity, whereas, in a mixture of ethanol–water (1:1), it decreased. The GBI@PPyNWs were successfully used to detect **Fe**^III^ in blood serum solution by changing from orange to dark red when the complex is formed. The assembly of PPy and GBI was proposed as biocompatible materials with potential use in biological detection to be used in the design of fluorescence probes for other metal ions.

**Scheme 8:**
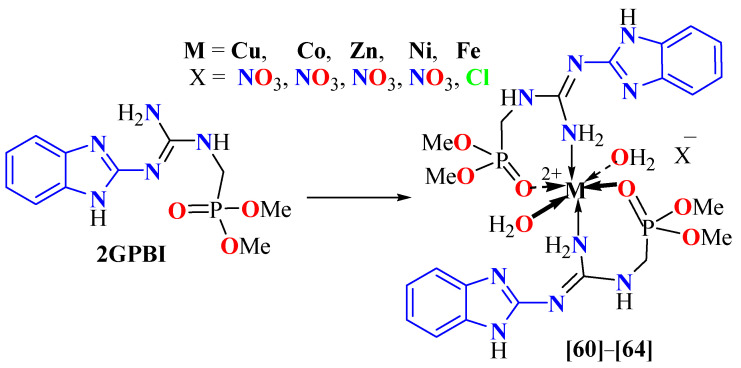
Coordination complexes **[M**(**2GPBI**)_2_**]**^2+^ derived from 2GPBI.

2GBI-phosphonate (2GPBI) coordination complexes **[M**(**2GPBI**)_2_**]**^2+^ (**M**^II^ = Cu, Co, Zn, Ni, Fe) **[60]–[64]** were synthesized, Scheme 8 [91] and characterised by standard methods. The metal ions were coordinated to the 2GPBI ligand through the guanidine nitrogen N12 and the P=O ending of the phosphonate to form seven-membered rings. Therefore, a six-coordinated octahedral/distorted octahedral geometry for all the complexes was suggested in Scheme 8.

**Figure 16:**
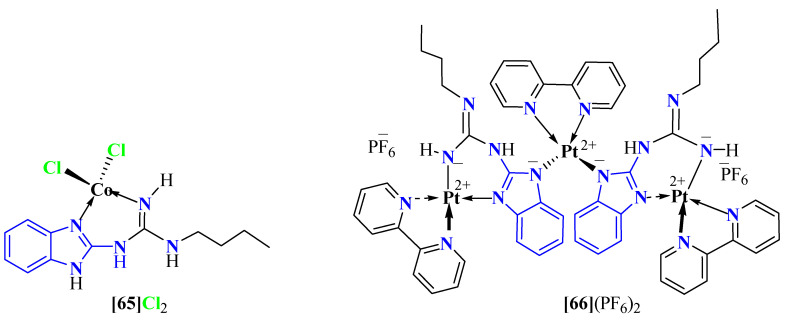
**Co**(*^n^***Bu_2_2GBI**)Cl_2_, [**65**Cl_2_] and [(**Pt**(2,2´-bipy))_3_(*^n^***Bu_2_GBI**)_2_](PF_6_)_2_, **[66]**(PF_6_)_2_.

The coordination complex **Co**(*^n^*Bu_2_**2GBI**)_2_Cl_2_ **[65**Cl_2_**]**, was synthesized with *N*-buthyl-2-guanidinebenzimidazole (*^n^*Bu_2_**2GBI**) ligand as a bright blue powder in 49% yield, as well as the coordination complex **[**(**Pt**(2,2’-bipy))_3_(*^n^***Bu_2_GBI**)_2_**]**(PF_6_)_2_, **[66]**(PF_6_)_2_, as a crimson powder in 51% yield, Figure 16 [92]. Complex **[66]**(PF_6_)_2_ showed unusual supramolecular properties, demonstrating a large self-assembly of **Pt**^II^ cations. Both complexes were tetracoordinate, with some self-recognition abilities due to their HB capabilities. The complexes exhibited bidentate coordination through the N3 and N12 nitrogen atoms of the *^n^*Bu_2_**2GBI**) ligands. **Pt**^II^ complex showed, through intermolecular HB and π-stacking, a possible supramolecular oligomer with a small cavity.

**Figure 17:**
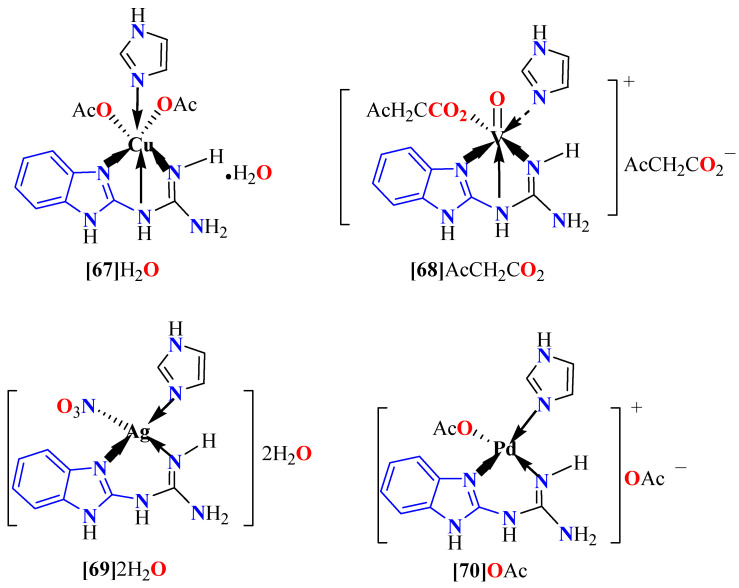
2GBI mixed ligand and its complexes **[67]**H_2_O, **[68]**AcCH_2_O_2_, **[69]**2H_2_O and **[70]**OAc.

The **Cu**^II^, **V**O^2+^, **Ag**^I^, and **Pd**^II^ mixed ligand coordination complexes **[67]**H_2_O, **[68]**AcCH_2_O_2_, **[69]**2H_2_O and **[70]**OAc were synthesized from 2GBI and imidazole, Figure 17 [93]. Their chemical structures were characterized by spectral, analytical and computational studies. Water molecules on the mononuclear complexes were identified by TGA analysis, and DFT studies of the complexes confirmed the proposed coordinating site and showed their optimal three-dimensional structures.

**Figure 18:**
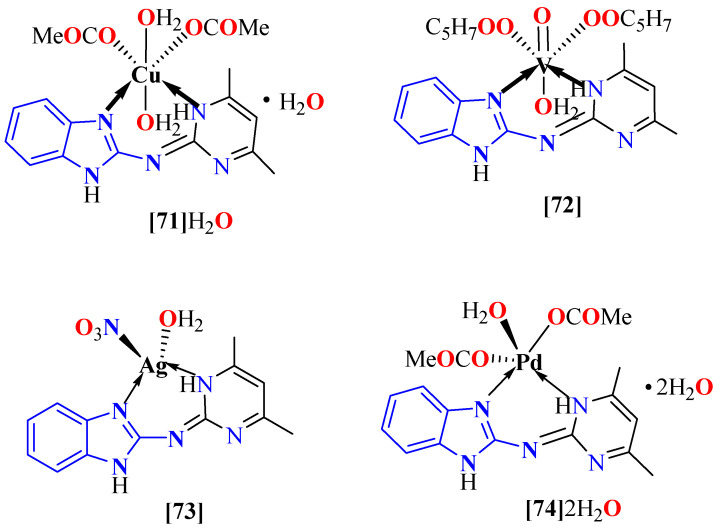
2GBI mixed ligand and its complexes **[71]**H_2_O, **[72]**, **[73]** and **[74]**2H_2_O.

Herein, the transition metal coordination complexes with DOBPA (4,6-dimethyl-N-(octahydro-2H-benzimidazol-2-ylidene)pyrimidin-2-amine) are included. This ligand was recently prepared by condensation between 2GBI and acetyl acetone with the aim of forming metallic catalysts [44]. The structure of DOBPA preserves the skeleton of 2GBI and introduces conformational restraints to favour complexation. Then, (DOBPA) was complexed with **Cu**^II^, **V**O^2+^, **Ag**^I^, and **Pd**^II^ through bidentate chelating mode to obtain the coordination complexes **[71]**H_2_O, **[72]**, **[73]** and **[74]**2H_2_O, Figure 18. Their structures, stability and stoichiometry of the complexes in solution were achieved using standard methods. The structure and electronic behavior of the complexes were supported by computational DFT methods, promising the **Pd**^II^ complex in the catalytic field. This complex was selected as a catalyst in the synthesis of 3-methyl-4-phenyl-4,9-dihydro-1H-pyrazolo[3,4-d][1,2,4]triazolo[1,5-a]pyrimidine derivatives using microwave irradiation in a one-pot reaction. The **Pd**^II^ complex **[74]**2H_2_O demonstrated superiority with high yield, short time, green conditions of reaction, catalyst recovery, and turnover.

## 6. Organo-Transition Metal Complexes (Organometallic Complexes)

Organometallic transition metal complexes bearing 2GBI are scarce; only **Hg**^II^, **Ru**^II,III^ and **Ir**^I,III^ complexes are known, all of them synthesized from a preformed organometallic compound. Most of them have been applied as catalysts in organic synthesis, taking advantage of the hydrogen bonding and tautomeric equilibria features of 2GBI.

**Figure 19:**
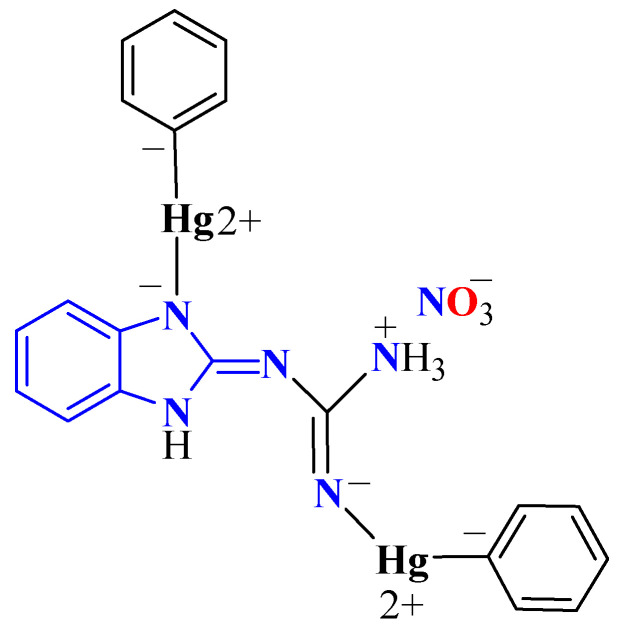
Proposed structure of the organo-mercury complex **[75]**NO_3_.

2GBI was reacted, as a monoprotic ligand, with basic phenylmercuric nitrate to produce a binuclear organo-mercury(II) complex of general formula **[**(C_6_H_5_·**Hg**(**2GBI**^2−^)(**Hg**·C_6_H_5_)**]**NO_3_, **[75]**NO_3_, Figure 19 [94]. The complex was insoluble in common organic solvents but was ionic in nitromethane solution. Comparison is made with other mercury–BI complexes.

**Figure 20:**
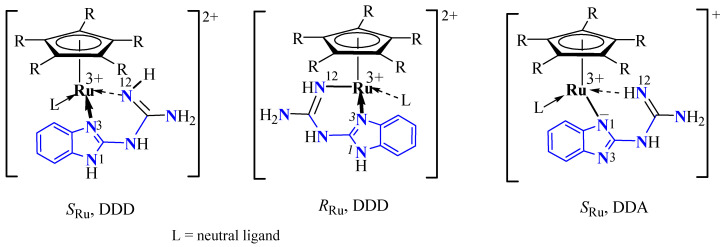
Chiral-in-metal 2GBI-Ru^III^ organometallic complexes.

The following section deals with chiral-in-metal 2GBI-**Ru** organometallic complexes. They are formed by a central **Ru**^III^ coordinated to cyclopentadienyl (Cp^−^) mono-anionic ligand that acts as a spectator ligand engaged in stabilising the organometallic complex. It is characterised by imposing a piano-stool geometry around the ruthenium center, which is chiral when there are four different ligands bound to it. Due to the non-symmetry of 2GBI, its bonding scheme is equivalent to two different substituents whose priorities for assigning the absolute configuration are N1,3 > N12 > Cp. 2GBI coordinates to **Ru**^III^ through N1 and N12, or N3 and N12, as anionic or neutral ligand, respectively. Coordination through N3 leads to a characteristic DDD HB pattern in the outer or second coordination sphere, whereas coordination through N1 and N12 leads to a DDA HB pattern, Figure 20.

**Scheme 9:**
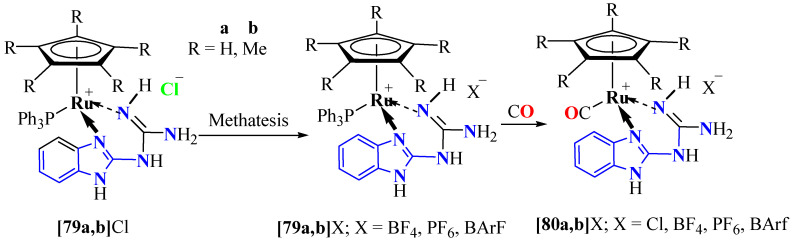
Synthesis of ruthenium organometallic complexes **[79a**,**b]**Cl, **[79a**,**b]**X and **[80a**,**b]**X; X^−^ = Cl, BF_4_, PF_6_, BArF.

**[**Cp**Ru2GBI**L**]**^2+^ type organometallic complexes were obtained by the reaction of **[**(η^5^-C_5_R_5_)**Ru**(PPh_3_)_2_**]**Cl, R = H **[76a]**Cl, and R = Me **[76b]**Cl, **[**(η^5^-C_9_H_7_)**Ru**(PPh_3_)_2_**]**Cl **[77]**Cl or **[**(η^5^-C_5_H_5_)**Ru**(CO)(NCCH_3_)_2_**]**PF_6_ **[78a]**PF_6_ and 2GBI in refluxing toluene as racemic mixtures. Then, the complexes **[**(η^5^-C_5_R_5_)**Ru**(PPh_3_)(**2GBI**)**]**Cl, R = H **[79a]**Cl, **[**(η^5^-C_5_R_5_)**Ru**(CO)(**2GBI**)**]**X, R = Me **[80b]**X, and indenyl organometallic complex **[**(η^5^-C_9_H_7_)**Ru**(PPh_3_)(**2GBI**)**]**Cl **[81]**Cl were prepared in 96%, 61–84% and 96% yields, respectively, Scheme 9 [95]. Several cationic complexes of formula **[79a]**X or **[80a]**X (X^−^ = Cl, BF_4_, PF_6_, BArF (BArF^—^ = B(3,5-C_6_H_3_(CF_3_)_2_)_4_)) can be obtained by metathesis of **[79a]**Cl or **[80a]**Cl salts in 77–87% yield. Reactions with CO gave the carbonyl organometallic complexes **[**(η^5^-C_5_H_5_)**Ru**(CO)(**2GBI**)**]**X **[80a]**X, in 87–92% yield. All organometallic complexes were characterized by (^1^H, ^13^C, ^31^P, ^19^F, ^11^B) NMR and 2D spectra assignments. X-ray diffraction structures of **[79a]**PF_6_**.**CH_2_Cl_2_ and**[79a]**BArF**.**CH_2_Cl_2_ was studied; the anion was hydrogen bonded to the cation in the former.

**Scheme 10:**
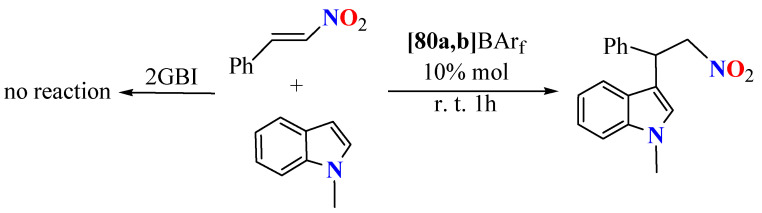
Catalyzed condensation reaction of indoles and *trans*-β-nitrostyrene.

The organometallic complexes **[80a,b]**X were evaluated as catalysts (10 mol %, RT) for the Friedel–Crafts alkylation reaction of indoles with *trans*-β-nitrostyrene, Scheme 10. There was a marked dependence of catalyst activities upon the cation. The chloride salts were not effective (0% yields, 31–48 h) and with X^−^ = BF_4_ and PF_6_ salts, the yields were poor (3–29%, 7–48 h); however, the BArF^−^ salts showed reactivities of 84% yield in 48 h and of 97% yield in 1.5 h. The nitro group of the functionalyzed indole forms a hydrogen bond with the GBI ligand. A BArF^−^ salt of methylated 2GBI **[***^N^*Me-**2GBI]**BArF was active (40% yield in 3.5 h) but much less than the BArF^−^ salts of the organometallic complexes **[80a,b]**^+^.

**Scheme 11:**
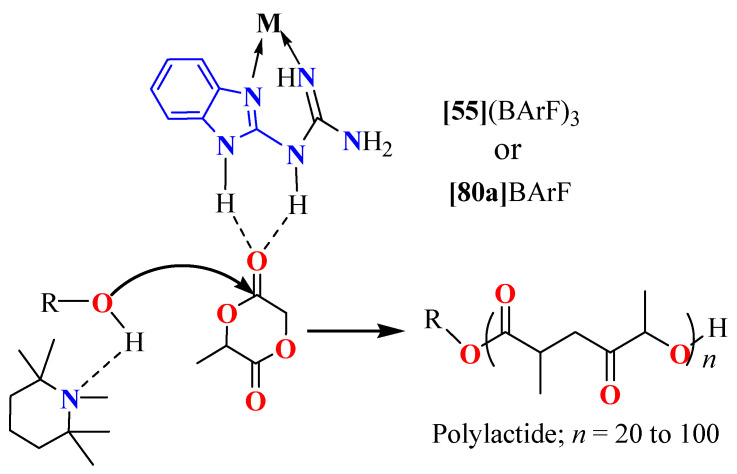
Catalyzed polymerisation reaction of *DL*-lactide.

The **Ru**^I^ organometallic complex **[80a]**BArF·1.5H_2_O and the **Co**^III^ coordination complex **[***mer*-**55]**(BArF)_3_·3H_2_O salts were evaluated as HB donor catalysts, Scheme 11 [96]. The addition of equimolar quantities of 1,2,2,6,6-pentamethylpiperidine (PMP) and 4-phenylbenzyl alcohol as hydroxylic initiator (**In**OH) controlled the ring-opening polymerisations of *dl*-lactide at loadings (1–3 mol %). These systems afforded polylactide with *M*_n_ values of 4000–11,000 g/mol and narrow dispersities (˂1.18). The MALDI-ToF mass spectra showed a series of peaks separated by *m*/*z* values of 144 and without transesterification side reactions between polymer chains. Runs with multiple charges of monomer established the living nature of the polymerisation, and ^1^H NMR or UV–visible experiments provided evidence for key HB interactions (**In**OH/PMP **[55]**(BArF)_3_/*dl*-lactide).

**Scheme 12:**
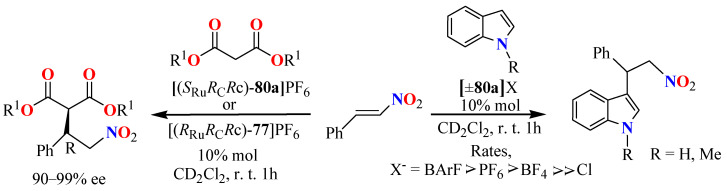
Application of 2GBI chiral cationic ruthenium complex as catalyst.

In order to explain the catalytic activity of the organometallic complexes **[80a]**X and **[55]**X_3_, their chiral-in-metal complexes bearing X^−^ = F, Cl, Br, I, BF_4_, PF_6_, BArF, were in silico modelled as effective HB donor catalysts in rationalising reactivity trends and enantioselectivities for several organic reactions, Scheme 12 [97]. Authors identified cation/anion HB motifs forming dyads or triads of *syn*-periplanar NH linkages. The gas phase ΔG values of each series followed the ΔG order Cl^−^ << BF_4_^−^ < PF_6_^−^ < BArF^−^, paralleling catalytic activities as the HB acceptor strengths diminish. With BF_4_^−^ and PF_6_^−^, all HBs involved F-A-F units (A = B, P); with BArF^−^ the isomers feature 2–6 NH∙∙∙FC^−^ and 1–2 NH∙∙∙π interactions.

**Scheme 13:**
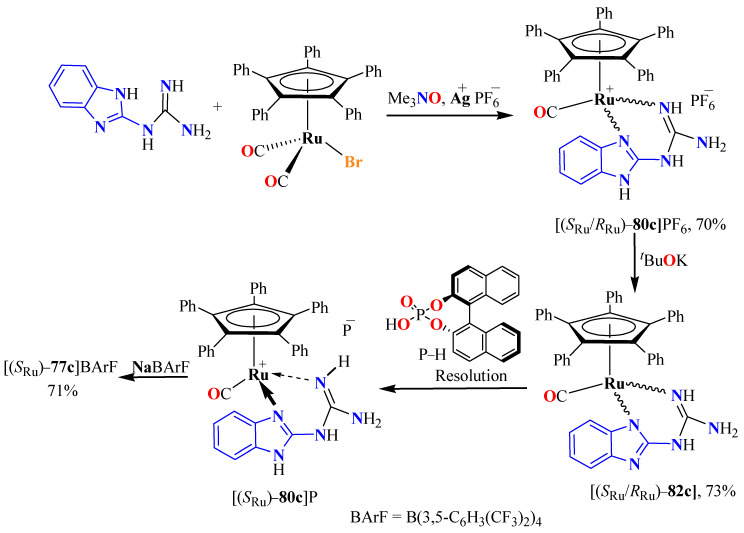
Enantioselective synthesis of the complex **[**(*S*_Ru_)-**80c]**BArF.

In other work, the steric effect of the organic ligand was increased by using a pentaphenylcyclopentadienyl ligand (C_5_Ph_5_^−^) in order to increase the Ru stereocenter effect on its stereoselectivity as a catalyst. Then, the treatment of the organometallic complex **[**(η^5^-C_5_Ph_5_)**Ru**(CO)_2_**]**Br **[80c]**Br with Me_3_NO, **2GBI**, and **Ag**PF_6_ gave **[**(η^5^-C_5_Ph_5_)**Ru**(CO)(**2GBI**)**]**PF_6_, **[80c]**PF_6_ in 70% yield or treatment of the complex [**80c**]PF_6_ with alumina, then NaBArF gave **[80c]**BArF in 69% yield, Scheme 13 [98]. Treatment of **[80c]**PF_6_with K*^t^*BuO deprotonates the 2GBI and the neutral organometallic complex [(η^5^-C_5_Ph_5_)**Ru**(CO)(**2GBI**)] [**82c**] was produced in 73% yield. Both cationic **[80c]**^+^ and neutral **[82c]** complexes were characterised by NMR and X-ray crystallography. The organometallic complex **[82c]** was protonated with enantiopure (*P*)-1,1′-binaphthyl-2,2′-diyl hydrogen phosphate (*P*-H) to yield the diastereomeric salts **[**(*R*_Ru_/*S*_Ru_)-**80c]***P* in 93%. The organometallic complex **[**(*S*_Ru_)-**80c]***P* was selectively precipitated from cold toluene/hexanes in 35% yield, and the subsequent addition of **Na**BArF yielded the complex **[**(*S*_Ru_)-**80c]**BArF in 71%. The absolute configuration was determined by circular dichroism (CD) spectroscopy.

The efficacy of the organometallic complex **[**(S_Ru_)-**80c]**BArF as a catalyst was tested in a series of reactions: (1) Friedel−Crafts alkylation of 1-methylindole with *trans*-β-nitrostyrene, (2) Michael addition of diethyl malonate to malononitrile or 2,4-pentanedione, (3) addition of thiophenol to Michael acceptors containing an oxazolidinone imide. In all cases, the enantioselectivities in all test reactions were very poor or not detectable; in addition, it was not practical to recover the catalyst [98].

**Scheme 14:**
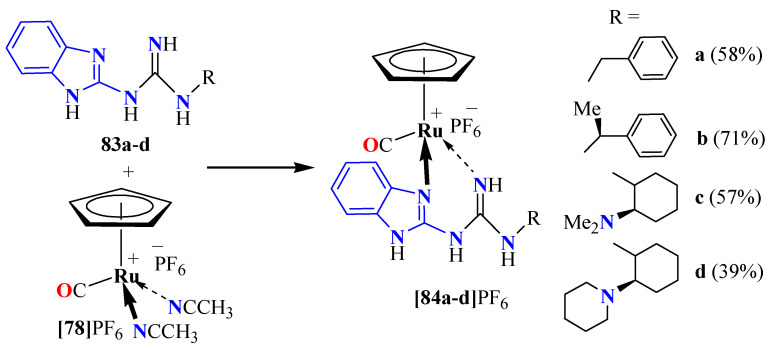
Synthesis of chiral **Ru**(II) organometallic complexes **[84a**–**d]**PF_6_.

In order to introduce chirality at the 2GBI ligand and test the influence of the chiral-in metal stereocenter in the ruthenium second sphere of coordination, *N*-alkyl-2GBIs (*^N^*R-2GBI), **83a**–**d**, were reacted with **[78a]**PF_6_ to afford the chiral-at-metal chelates **[**(η^5^-C_5_H_5_)**Ru**(CO)(***^N^*R-2GBI**)**]**PF_6_, **[84a**–**d]**PF_6_, in 39–77% yield, Scheme 14 [99]. The **Ru**,C configurational diastereomers of the complex **[84c]**PF_6_ were (*R*_Ru_*R*_C_*R*_C_ > 99:01dr; *S*_Ru_*R*_C_*R*_C_ < 2:98 dr), which in solution at room temperature slowly epimerised at ruthenium. Configurations were assigned by CD spectra and with the crystal structure of **[**(*R*_Ru_*R*_C_*R*_C_)-**84c]**(Δ)-TRISPHAT (TRISPHAT = P(*o*-C_6_Cl_4_O_2_)_3_), which showed **GBI**-RNH HBs with the TRISPHAT oxygen atoms. The salt consisted of a 50:50 mixture of both diastereomers of the ruthenium cation (R_Ru_R_C_R_C_/S_Ru_R_C_R_C_) and both enantiomers of the TRISPHAT-anion (Δ/Λ).

**Scheme 15:**
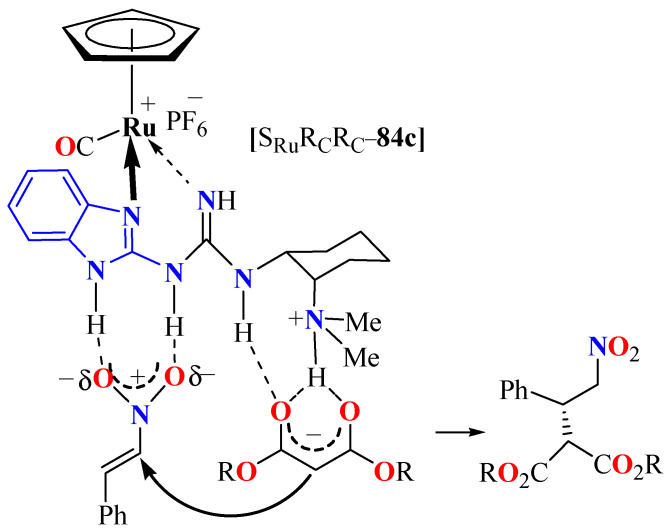
Model proposed for the addition of malonate esters to *trans*β-nitro-styrene catalyzed by the complex **[**(S_Ru_R_C_R_C_)-**84c]**.

The use of 10 mol % of both diastereomers of the organometallic complex **[**(*R*_Ru_*R*_C_*R*_C_)-**84c]**(Δ)-TRISPHAT catalyzed the additions of malonate esters to nitroalkenes at room temperature in high yields with enantioselectivities of 90–99% ee for aryl-substituted nitroalkenes. The dominant product configurations were identical, showing the chiral ruthenium center to have little influence. The free ***^N^*R**-**2GBI** ligand exhibited a modest activity. Unlike most transition-metal catalyzed reactions, there is no direct interaction of the substrate with the ruthenium; rather, evidence is presented for second coordination sphere promoted catalysis mediated by HBs derived from two NH groups of ***^N^*R-2GBI** that chelation pre-organises in a *syn-*periplanar conformation, Scheme 15.

Organometallic complexes **[**(*R*_Ru_*R*_C_*R*_C_)-**84c]**BArF and **[**(*S*_Ru_*R*_C_*R*_C_)-**84c]**BArF were resolved. Both were tested as an effective HB donor catalyst for asymmetric induction in the catalysis of the additions of 1,3-dicarbonyl compounds to nitroalkenes. **[**(*S*_Ru_*R*_C_*R*_C_)-**84c]**PF_6_ was the more reactive catalyst, although both diastereomers gave the product with the same configuration in the created stereocenter, Table 4 [99].

The mechanism and basis for enantio-selection of the **[**(*S*_Ru_*R*_C_*R*_C_)-**84c]**PF_6_ and **[**(*R*_Ru_*R*_C_*R*_C_)-**84c]**PF_6_ enantiopure chelate salts in the catalysis of the additions of 1,3-dicarbonyl compounds to nitroalkenes were supported by DFT calculations [100]. Such chelate salts were compared with the parent 2GBI organometallic complexes **[**(*S*,*R*)_Ru_-**80a]**PF_6_, using trimethylamine as a base. The di-carbonyl compound forms a hydrogen bonding with the NHs of the 2GBI ligand, forming transition states almost equal in energy. In contrast, after similar bonding of the di-carbonyl compound to the organometallic complex **[**(*S*_Ru_*R*_C_*R*_C_)-**84c]**PF_6_ or **[**(*R*_Ru_*R*_C_*R*_C_)-**84c]**PF_6_, a proton is transferred to the NMe_2_ moiety, giving an enolate and a HNMe_2_^+^ group. The latter mediates the introduction of *trans*-β-nitro-styrene such that one enolate π face attacks the C*_si_*=C*_re_*Ph face to give an addition product with an *R* configuration, in agreement with the experiment. Thus, DFT calculations predict the configurations of the catalyst carbon stereocenters control the product stereochemistry, in good agreement with results experimentally obtained, Table 4. Additions of 2,4-pentanedione to trans-β-nitrostyrene, catalyzed by the organometallic complexes **[**(*S*_Ru_*R*_C_*R*_C_)-**84c]**PF_6_ and **[**(*R*_Ru_*R*_C_*R*_C_)-**84c]**PF_6_, afforded (*R*)-3-(2-nitro-1-phenylethyl)pentane 2,4-dione in 70–75% yields and >99% ee [100]. This result confirmed that the product configuration is controlled by the carbon configurations of the catalyst, whereas the ruthenium configuration has virtually no influence.

**Scheme 16:**
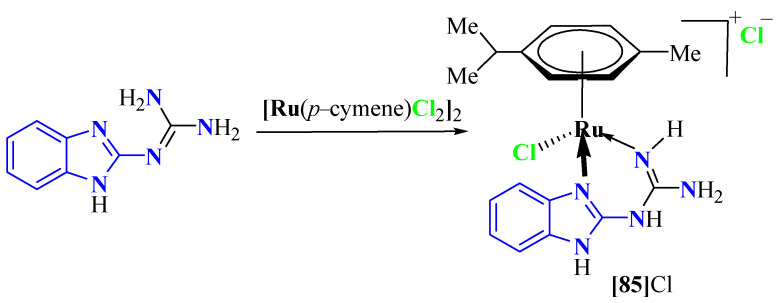
Synthesis of the neutral organometallic complex [Ru(*p*-cymene)(2GBI)Cl]Cl, **[85]**Cl.

The last ruthenium organometallic complex considered in this section is the complex **[Ru**(*p*-cymene)(**2GBI**)Cl**]**Cl **[85]**Cl, obtained from the reaction between **2GBI** in MeOH and **[Ru**(*p*-cymene)Cl_2_**]**_2_ in dichloromethane, as an orange crystalline solid in 79% yield, Scheme 16 [101].

**Scheme 17:**
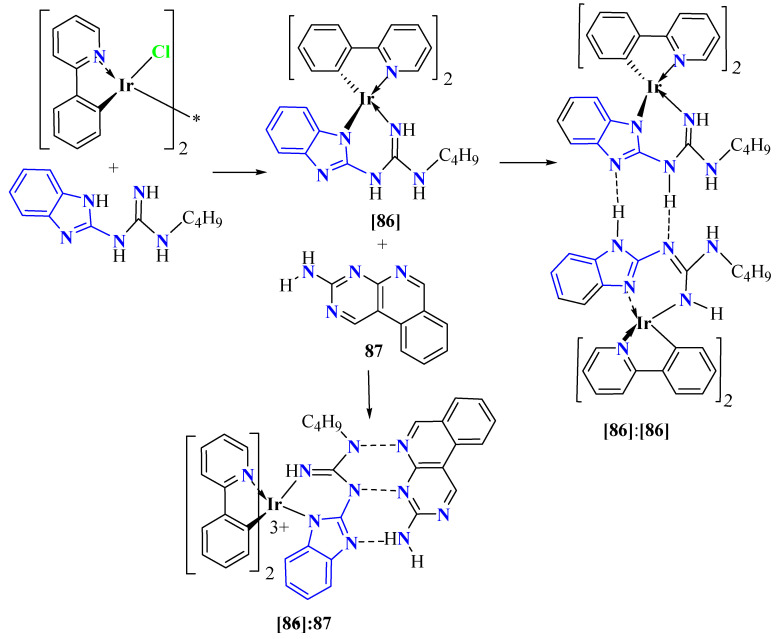
Formation of neutral Ir(III) organometallic complex **[86]**, its HB supramolecular assembly **[86]**:**[86]** and triple HB supramolecular assembly **[86]**:**87** system.

***^N^*Bu**-**2GBI** was reacted with iridium μ-chloro-bridged dimer **[Ir**(ppy)_2_Cl**]**_2_ (ppyH = 2-phenylpyridine) in toluene in the presence of potassium carbonate to give the neutral **Ir**(III) organometallic complex **[Ir**(2-ppy)_2_(***^N^*Bu**-**2GBI**)**] [86]** as yellow solid in 44% yield [102]. The complex crystallised as an HB dimer **[86]**:**[86]**, Scheme 17. The dimeric assembly exhibited green photoluminescence (PL) with emission maxima at 491 and 518 nm with 20% Φ_PL_ (quantum yield) in DCM solution and 34% in 5 % wt PMMA doped film. In the dimmer, two ppyH ligands were bonded via bidentate C and N bonding to each iridium atom with the nitrogen atoms in a *trans* configuration and with the guanidine ligand as a 6-membered N,N-chelate. The organometallic complex **[86]** was associated with pyrimido-[4,5-c]isoquinolin-3-amine **87** in a mixture of CHCl_3_/DMSO 98:2. The formed supramolecular assembly **[86]**:**87** revealed an association constant of Ka = 4.3 × 10^3^ M^−1^ and was described by a contiguous AAD–DDA array. The triple HB **[86]**·**87** heterodimeric system exhibited modulated photophysical properties compared with the corresponding mononuclear organometallic complex **[86]**.

The HB supramolecular assembly **[86]**:**87** showed electrochemiluminescence (ECL) due to the HB interaction of the coordinated 2GBI in the **Ir**^III^ complex **[86]** and the ligand pyrimido-[4,5-c]isoquinolin-3-amine, **87** [103]. ECL was demonstrated through cyclic voltammograms (CVs), and the HB supramolecular assembly **[86]**:**87** displayed a reduction in contrast to the inexistent reduction at organometallic complex **[86]** and compound **87**. Benzoate peroxide (BPO), as a co-reactant, was used to form the exciplex [PhCO_2_^−^:**[86]**] and excimer **[86]**:**[86]**. Furthermore, benzoate radical PhCO_2_^−^ also reacted with the HB supramolecular assembly **[86]**:**87** to form [PhCO_2_^−^·**[86]**]:**87** and generate the same ECL emission as PhCO_2_^−^:**[86].** Insights into the ECL processes of this HB complex were provided.

**Figure 21:**
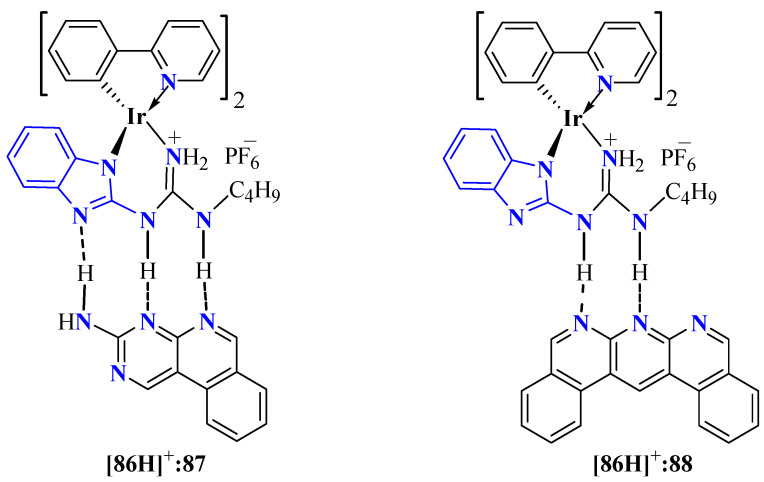
Binding of complex **[86**H**]** with partners **87** and **88**.

The organometallic complex **[86**H**]**PF_6_ was synthesized from **[86]**Cl by ligand exchange using potassium hexafluorophosphate KPF_6_ as the source of PF_6_^−^ counterion, Figure 21 [104]. Organometallic complex **[86**H**]**^+^ was hydrogen bonded with partners **87** and benzo[f]isoquinolino[3,4-b][1,8]naphthyridine **88**, **[86**H**]**^+^:**87**/**88**, to obtain their association constant by using UV–vis absorption spectroscopy titration methods. Titration study for supramolecular assembly **[86**H**]**^+^:**87**, Figure 20 revealed an increased association constants K_11_ = 1.1 × 10^6^ M^−1^ and K_12_ = 2.1 × 10^3^ M^−1^ (UV–vis, CHCl_3_/DMSO, (99:1 *v*/*v*)) in comparison to neutral supramolecular assembly **[86]**:**87**. The association strength for protonated supramolecular assembly **[86**H**]**^+^:**87** (DDD^+^–AAA array) where protonation of the 2GBI would lead to a DDD^+^ system, a perfect complement to **87**^−^ did not increase compared to neutral supramolecular assembly **[86]**:**87** (DDA–AAA array).

## 7. Biological Activities of 2GBI Complexes

In this last section, the known biological activities of the main group, transition metals and organometallic complexes of 2GBI are analyzed. A summary of the biological performances is given in Table 5. Antimony complexes are first mentioned, followed by transition metal complexes and organometallic complexes.

Antimony complexes **[37a,b]** were prepared for biological purposes [69]; they exhibited glutathione reductase (GR) inhibitory activity with IC_50_ value in the range of 4.87–5.37 μM, being the best inhibitors compared with other antimony complexes bearing ligands such as 2-benzyl-2-thiopseudourea, triazine, pyrimidines and pyridines. GR inhibitor activity of ligands decreases as follows: 2GBI > 2-benzyl-2-thiopseudeourea > triazine > 2-aminopyrimidine > 2-aminopyridine > 5-methylpyridine > 4,6-methoxypyrimidine. Complexes **[37a,b]** act as metalonucleases for double-strand cleavage of DNA.

The anti-leishmanial activities of complexes **[37a,b]** were assessed in vitro against *Leishmania tropica* promastigotes [105], being **[37a**] the best complex showing 3.2% growth inhibition at a concentration of 31.3 μg/mL, compared with other antimony complexes of formula **[Sb**L_n_Cl_3_**]** (where n= 1 or 2, L = substituted 2-aminopyridines, 2-aminopyridines, 2-pyrrolidines and other heterocycles.

Complexes **[54]**Cl(**Zn**Cl_4_)·CH_3_OH and **[54]**Cl_3_·4H_2_O inhibit ATP synthesis and electron flow in spinach thylakoids, behaving as Hill reaction inhibitors [81]. Complex **[54]**Cl(**Zn**Cl_4_)·CH_3_OH targeted the Secondary Quinone acceptor (Q_B_), completely inhibiting PSII, whereas complexes **[54]**Cl_3_ and **[55]**Cl_3_ acted in the span from P_680_ to Primary Quinone Acceptor (Q_A_) and at the b_6_f complex. The most potent complex was **[54]**Cl(**Zn**Cl_4_), whose efficiency was the result of synergy between **[54]** and the (**Zn**Cl_4_)^2−^. The IC_50_ values of complex **[54]**Cl(**Zn**Cl_4_) were 258 μM for noncyclic photosynthetic photophosphorylation, 200 μM for uncoupled electron transport, and 95 μM for uncoupled photosystem II electron flow.

The synthesized **Cu**^II^ neutral complex **[38**Cl_2_**]**, [**38**Br_2_]·H_2_O and **[39]**(NO_3_)_2_·4H_2_O, the **Co**^II^ complexes **[40]**(NO_3_)_2_·4H_2_O, **[40**Cl_2_**]**∙2H_2_O and **[40**Br_2_**]**·H_2_O and the **Zn**^II^ complexes [**42**Cl_2_], [**42**Br_2_], and **[43]**(NO_3_)_2_ · H_2_O were evaluated to assess their cytotoxic activity using human cancer cell lines, PC3 (prostate), MCF-7 (breast), HCT-15 (colon), HeLa (cervical-uterine), SKLU-1 (lung) and U373 (glioblastoma) [73]. The Cu^II^ complex **[38**Br_2_**]**·H_2_O showed considerable cytotoxic activity on HeLas cells with IC_50_ = 115.3 μM. The cytotoxic activity was related to the halides in the coordination sphere of the metal ion. Other ligands were also tested such as 2-R-benzimidazoles R = Me, Ph, Cl, NCO_2_Me), the most active complex **[**Cu(2cmbz)Br_2_**]**0.7H_2_O 2cmbz = 2-benzimidazolecarbamate with IC_50_ = 26.7 μM on HCT-15 cells.

The **Co**^II^ complex **[41**Cl_2_**]** 2H_2_O was synthesized from the reaction of dried **Co**Cl_2_ salt with 2GBI in hot ethanol [106] and tested for **Fe**^II^ chelation and antioxidants. **[41**Cl_2_**]** 2H_2_O showed efficacy at 10, 20 and 40 μM (*p* < 0.005), compared with the reference, EDTA. At 50 μg/mL, 95% of iron chelation was observed, the complex inhibited 85 % by DPPH scavenging potential and 61% of efficacy in the Fenton reaction was observed. The IC_50_ values of the complex were 25, 29 and 41 µg/mL against **Fe**^II^ chelation, DPPH, and hydroxyl quenching assays. It was proposed that the nitrogen-containing moieties in the complex could contribute to the high antioxidant potential; however, further molecular studies to decrypt the biochemical mechanism of action are needed.

The binding affinity of the complexes **[67]**H_2_O, **[68]**AcCH_2_O_2_, **[69]**2H_2_O and **[70]**OAc with DNA were tested through agarose gel, electronic spectroscopy and viscosity measurements [93]. A calf thymus DNA test was used. The complexes bind with good affinity to CT-DNA electrostatically through exterior contact, replacement, intercalation and groove surface. In general, the values of the binding constants to CT-DNA (K_b_) follow the order: **[70]**OAc > **[69]**2H_2_O > **[68]**AcCH_2_O_2_ > **[67]**H_2_O > 2GBI.

In vitro antibacterial, antifungal, cytotoxic and antioxidant activities were tested for the complexes. The antimicrobial behavior of the tested compounds against different bacterial (*S. typhimurium* (−ve), *E. coli* (−ve), *B. cereus* (+ve)) and fungal (*A. flavus*, *G. candidum*, *F. oxysporum*) classes has been estimated employing the disc diffusion technique. The minimum inhibition zone was measured and expressed in % compared with the results obtained with ofloxacin. The antimicrobial activity of studied compounds is ordered as follows: **[70]**OAc > **[69]**2H_2_O > **[67]**H_2_O > **[68]**AcCH_2_O_2_ > 2GBI [93].

Hep-G2 (liver cancer), MCF-7 (breast cancer) and HCT-116 (colon cancer) cell lines were used for cytotoxicity. The IC_50_ values (μg/μL) of all compounds were similar to doxorubicin (dox), used for comparison purposes. The cytotoxic activity against tumoral cells is ordered as follows: dox > **[70]**OAc > **[69]**2H_2_O > **[67]**H_2_O > **[68]**AcCH_2_O_2_ >> 2GBI. Molecular orbital calculations and docking studies indicated promising inhibitory features of the Pd complex **[70]**OAc, which are in agreement with in vitro results [93].

Complex **[85]**Cl and the analogous benzoxazole (2GBO) and benzothiazole (2GBS) complexes were studied in their interaction with calf thymus DNA, revealing that the complexes are better DNA binders than the free ligands [101]. The authors explain this improvement to the imposed planarity of 2GBI by ruthenium coordination, enabling it to serve as a better intercalator. Partial intercalation of complexed 2GBI, 2GBO and 2GBS ligands and electrostatic and HB interactions with DNA were proposed by docking. The complex **[85]**Cl was the most cytotoxic in PC3 (prostate cancer) cell line (IC_50_ = 40 ± 2 μM) and A549 (lung cancer) cell line (IC_50_ = 69 ± 1 μM), showing comparable activity to cisplatin. On the other hand, **[85]**Cl was found to be non-toxic to the BPH1 (human prostate benign tumor) cell line. The tests were performed using the 3-(4,5-dimethylthiazol-2-yl)2,5-diphenyltetrazolium bromide) (MTT) assay. Besides, it was demonstrated that cells die by apoptosis, and the complex can be traced in cells due to their prominent fluorescent emissive nature.

The antiproliferative activity of the iridium complex **[86]** was also tested on human ovarian cancer cell lines EFO-21, EFO-27 and COLO-704 and their cisplatin-resistant sublines, as well as in COLO-704rCDDP1000 using the MTT assay [107]. The *^N^*Bu-2GBI ligand showed toxicity in all cell lines, with IC_50_ values in the low micromolar range of 10.1 μM, whereas the iridium complexes **[86]** had an average IC_50_ value of 1.4 μM, being much more active than the free 2GBI ligand and the analogous iridium complex with thiourea ligand. Then, the effect of the metal fixing the conformation is evident in the antiproliferative activity. The strong fluorescent emission of **[86]** allowed us to track it in living cells. Then, it was demonstrated that the endoplasmic reticulum is the target of complex **[86]**.

## 8. Conclusions

2-guanidinobenzimidazole results in the attachment of the guanidine group to the C2 of a BI ring. This junction extends the electronic conjugation of the BI ring to the exocyclic guanidine group, making 2GBI a fully conjugated planar molecule. The five nitrogen atoms with free lone pairs and the five labile hydrogen atoms make this kind of compound amphoteric in character. The molecule is considered as a super base in a dynamic tautomeric equilibrium. These characteristics make this class of compounds helpful in hydrogen bonding and coordinating bond formation. All tautomeric structures of 2GBI show an intramolecular N12–H∙∙∙N3 hydrogen bond; this interaction makes the N1, N10 and N13 can act as DDD, DDA, DAD and ADA hydrogen bonding motifs in host–guest complexation. Also, these motifs can form labile complexes with biological receptor sites. The protonation and methylation reactions of 2GBI showed the N3 as the preferred base site, demonstrating its coordination capacity as a chelate. The same behavior of N3 was demonstrated in reactions with boron derivatives.

In medicinal chemistry, the 2GBI is a very important molecule because its biological activities found that just as a stimulator, an inhibitor of the transport of Na^+^ and K^+^ in the apical membrane of the skin, diminishes the gastric acid secretion, acts as hypoglycaemic, hypotensive and recently, 5-cloro-2GBI was found to be considered a non-selective inhibitor of the human hHv1 channel.

On the other hand, it is known that metal ions play an essential role in several biological processes, and the interaction of metals with biologically active ligands, just as in drugs, has been a topic of great interest. In this sense, it is interesting to see the development of 2GBI-based complexes with transition and main group metals that could improve the biological activities of 2GBI. For instance, platinum metal-based coordination and organometallic complexes have been widely used as chemotherapeutics in fighting cancer, and since ruthenium can bind DNA in several possible ways, they are promising candidates for developing novel anticancer agents. Hence, the ruthenium-based species could be used to treat platinum-resistant cancers.

In general, 2GBI acts as a monodentate and bidentate ligand and forms mono-bis and -tris complexes with various metals. The mode of coordination is through N3 and N12, forming a six-membered ring. With one chelating 2GBI ligand, the structure was either tetrahedral or octahedral. With two chelating ligands, a square-planar geometry was stabilised. In contrast, three 2GBI bi-dentated ligands were found in octahedral structures. It is interesting to observe that, 2GBI being such a strong chelating ligand, in some cobalt compounds, monodentate coordination through the imidazole N3 is preferred over the chelate, and the structure results are tetrahedral.

In the transition metal coordination complexes of 2GBI, formally, one of the two protons on the N12 is substituted by the metal atom and coordinated by the imidazole N3. In this case, 2GBI behaves as a monoanionic ligand. Nevertheless, the resulting structure is the average of two resonance contributors.

Another common bonding pattern of 2GBI to the metal is through two coordination bonds, in such cases also by N3 and N12 nitrogen atoms, behaving as neutral ligands. In the case of the complexes with two 2GBI chelating ligands, two complementary DAD motifs were generated on each ligand. Therefore, the complex behaves as an HB unit that can form 1:2 adducts with complementary ADA hydrogen-bonding organic compounds. The resulting complexes were applied in the treatment of contaminated water, and they exhibited good cytotoxic activity against cancer cells and good antioxidant activities.

Chiral organometallic ruthenium complexes generated metal-templated HB donors as organo-catalysts for carbon–carbon bond formation reactions with excellent asymmetric induction where the second coordination sphere promoted catalysis mediated by HBs derived from two NH groups of 2GBI, which pre-organised the conformation.

## Figures and Tables

**Table 1:** 2-Guanidinobenzimidazole derivatives from Brönsted acids and methyl iodide.

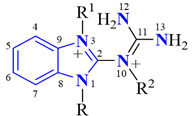												
**Substituent**	**Compounds**											
	**1a**	**1b**	**1c**	**1d**	**1e**	**1f**	**1g**	**1h**	**1i**	**1j**	**1k**	**1l**
R	H	Me	H	H	H	H	H	H	H	Me	Me	Me
R^1^	LP	LP	H	H	H	H	H	**B**H_3_	LP	H	**B**H_3_	Me
R^2^	LP	LP	LP	H	LP	LP	LP	LP	H	LP	LP	LP
Anion	---	---	H**SO**_4_^−^	**S** **O**_4_^2−^	**B**F_4_^−^	**Cl** ^−^	**Cl** **O**_4_^−^	---	Ac**O**^−^	**Cl** ^−^	---	**I** ^−^

LP = lone pair.

**Table 2:** Metal complexes of 2GB with 1:1 and 1:2 stoichiometries **M**^II^ complexes (**M**^II^ = **Cu**, **Co**, **Zn, Ni**) and counterions.

Formula	Num.	Ligands/Anions∙nH_2_O			
**Cu**(**2GBI**)^2+^	**38**	**[** **38** **Cl**_2_ **]**	**[** **38** **Br**_2_ **]**		
**Cu**(**2GBI**)_2_^2+^	**39**	**[39]**∙H_2_**O**	**[39Br**_2_**]**∙2H_2_**O**	**[39]**(**NO**_3_)_2_∙H_2_**O**	**[39]**(Ac**O**)_2_∙H_2_**O**
**Co**(**2GBI**)^2+^	**40**	**[40Cl**_2_(H_2_**O**)_2_**]**∙5H_2_**O**			
**Co**(**2GBI**)_2_^2+^	**41**	**[41Cl**_2_**]**∙3H_2_**O**		**[41**(H_2_**O**)_2_**]**(**NO**_3_)_2_∙4H_2_**O**	**[41**(Ac**O**)_2_**]**∙2H_2_**O**
**Zn**(**2GBI**)^2+^	**42**	**[42Cl**_2_**]**∙H_2_**O**	**[42Br**_2_**]**∙H_2_**O**		
**Zn**(**2GBI**)_2_^2+^	**43**			**[43]**(**NO**_3_)_2_∙H_2_**O**	
**Ni**(**2GBI**)_2_^2+^	**44**	**[44Cl**_2_**]**∙H_2_**O**	**[44Br**_2_**]**∙3H_2_**O**	**[44]**∙X_2_	

X^−^ = **I**, **NO**_3_, Ac**O.**

**Table 3:** Metal complexes of 2GBI with 1:2 and 1:3 stoichiometries (M^III^ = Rh, Ir) and counterions.

Formula	Comp.	Anions·nH_2_O			
**Rh**(**2GBI**)_2_^3+^	**47**	**[47X**_2_**]**X	**[47Cl**_2_**]NO**_3_·4H_2_**O**	**[47**(H_2_**O**)_2_**]Cl**_3_·H_2_**O**	**[47**(H_2_O)_2_**]**(**NO**_3_)_3_·4H_2_**O**
		**[47**(**N**H_3_)_2_**]Cl**_3_	**[47**(py)_2_**]Cl**_3_	**[47**(pic’)**]SO**_4_·4H_2_**O**	**[47**(bipy)**]**I_3_
		**[47**(pic’)**]Cl**_2_·3H_2_**O**,	**[47**(bipy)**]Cl**_3_	**[47**(*o*-phen)**]Cl**_3_	**[47**(*o*-phen)**]**I_3_
**Rh**(**2GBI**)_3_^3+^	**48**	**[48]** **Cl**_3_	**[48]**(**ClO**_4_)_3_		
**Ir**(**2GBI**)_2_^3+^	**49**	**[49X**_2_ **]X**	**[49** **Cl**_2_ **]** **Cl** **O**_4_	**[49**(**N**H_3_)_2_**]Cl**_3_	**[49**(Py)_2_**]Cl**_3_
		**[49**(bipy)**]Cl**_3_	**[49**(*o*-phen)**]Cl**_3_	**[49**(H_2_**O**)_2_**]Cl**_3_·2H_2_**O**	

X^−^ = **Cl**, **Br**, **I**, **NO**_3_, **N**C**S**.

**Table 4:** Enantioselective catalysis with diastereomeric bifunctional chiral-ruthenium organometallic complexes **[84c]**BAr_f_, yields (%Y), and enantiomeric excess (%ee) are given.

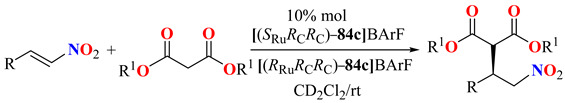								
R	Ph	3,5-Cl_2_Ph	3,5-CH_2_O_2_Ph	2-Furanyl	Ph	Ph	Ph	CH_3_(CH_2_)_5_
R^1^	Et	Et	Et	Et	*^i^*Pr	Me	Me	Et
*S*_Ru_-84**c**BAr_f_								
%Y	95	99	83	85	*75*	97	70	49
%ee	93	93	91	99	*91*	91	99	83
*R*_Ru_-84**c**BAr_f_								
%Y	92	96	84	74	*70*	95	75	41
%ee	91	98	89	99	*91*	9	99	87

**Table 5:** Studied biological performances of coordination and organometallic complexes of 2GBI.

Complex	Biological Activity	Quantitative Parameter	Observations	Ref.
**[37a,b]**	Inhibitors of glutathione reductase (GR)	IC_50_ (μM) = 5.37 **[37a]**, 4.87 **[37b]**	Acts as metalonucleases for double-strand cleavage of DNA	[69]
**[37a]**	Anti-leishmaniasis	3.2% growth inhibition at 31.3 μg/mL	Compared with other Sb complexes with heterocyclic ligands	[105]
**[54]**Cl(ZnCl_4_)	Inhibit ATP synthesis	IC_50_ (μM) = 258	Noncyclic photosynthetic photophosphorylation Inhibit ATP synthesis	[81]
		IC_50_ (μM) = 200	Uncoupled electron transport	[81]
		IC_50_ (μM) = 95	Uncoupled photosystem II electron flow	[81]
**[38**Br_2_**]∙**H_2_O	Antiproliferative	48% survival at 10 μg/mL	PC3 (prostate)	[73]
**[39]**(NO_3_)_2_·4H_2_O	Antiproliferative	85% survival at 10 μg/mL	PC3 (prostate)	[73]
**[40**Cl_2_·2H_2_O**]**	Antiproliferative	76% survival at 10 μg/mL	U373 (glioblastoma)	[73]
**[43]**(NO_3_)_2_∙H_2_O	Antiproliferative	54% survival at 10 μg/mL	HCT-15 (colon)	[73]
**[39**Br_2_**]**	Antiproliferative	IC_50_ (μM) = 115.3	HeLa (cervical-uterine)	[73]
**[41**Cl_2_**]**∙(H_2_O)_2_	Antioxidant	25 µg/mL, Fe(II) chelation; 29 µg/mL DPPH; 41 µg/mL hydroxyl quenching	Compared with EDTA	[106]
**[67]**H_2_O **[68]**AcCH_2_O_2_ **[69]**2H_2_O **[70]**OAc	DNA binding	K_b_ = 0.113 × 10^4^–2.03 × 10^4^	Calf-thymus model	[93]
	Antibacterial	%A.I. = 82.9–95.6 (*S.T.*), 84.7–94.9 (*E.C.*), 88.1–95.7 (*B.C.*)	Compared with ofloxacin *S.T.*, *S. typhimurium; E.C.*, *E. coli; B.C.*, *B. cereus*	[93]
	Antifungal	%A.I. = 79.2–93.2 (*A.F.*), 88.4–95.4 (*G.C.*) 81.2–93.4 (*F.O.*)	Compared with ofloxacin *A.F.*, *A. flavus*; G.C., *G. candidum;* F.O., *F. oxysporum*	[93]
	Antiproliferative	IC_50_ (μg/μL) = 6.5–10.1 MCF7; 6.3–11.1 (HCT-116); 4.95–8.75 (Hep-G2)	Compared to dox: IC_50_ (μg/μL) = 5.0–6.4; Compared to 2GBI: IC_50_ (μg/μL) = 20.4–26.6 Cancer cell lines: MCF7, breast; HCT-116, colon; Hep-G2, liver	[93]
**[85]**Cl	DNA binding	K_b_ = 1.6 × 10^3^	Calf thymus model	[101]
	Antiproliferative	IC_50_ (μM): 40 ± 2 (PC3); 263 ± 2 (BPH1) 69 ± 1 (A549); 70 ± 3 (WI-38)	Cancer cell lines: PC3, prostate; BPH1, human benign prostate tumor; A549, lung cancer; WI-38, normal lung fibroblast. Comparable activity like cisplatin	[101]
**[86]**	Antiproliferative	IC_50_ (μM): 1.4	Human ovarian cancer cell lines EFO-21, EFO-27 and COLO-704 and their cisplatin-resistant sublines. Compared with free *^N^*Bu-2GBI: IC_50_ (μM) = 10.1	[107]
